# Sediment subduction in Hadean revealed by machine learning

**DOI:** 10.1073/pnas.2405160121

**Published:** 2024-07-08

**Authors:** Jilian Jiang, Xinyu Zou, Ross N. Mitchell, Yigang Zhang, Yong Zhao, Qing-Zhu Yin, Wei Yang, Xiqiang Zhou, Hao Wang, Christopher J. Spencer, Xiaocai Shan, Shitou Wu, Guangming Li, Kezhang Qin, Xian-Hua Li

**Affiliations:** ^a^State Key Laboratory of Lithospheric and Environmental Coevolution, Institute of Geology and Geophysics, Chinese Academy of Sciences, Beijing 100029, People’s Republic of China; ^b^College of Earth and Planetary Sciences, University of Chinese Academy of Sciences, Beijing 100049, People’s Republic of China; ^c^Key Laboratory of Mineral Resources, Institute of Geology and Geophysics, Chinese Academy of Sciences, Beijing 100029, People’s Republic of China; ^d^Key Laboratory of Computational Geodynamics, College of Earth and Planetary Sciences, University of Chinese Academy of Sciences, Beijing 100049, People’s Republic of China; ^e^Key Laboratory of Earth and Planetary Physics, Institute of Geology and Geophysics, Chinese Academy of Sciences, Beijing 100029, People’s Republic of China; ^f^State Key Laboratory of Lunar and Planetary Sciences, Macau University of Science and Technology, Macau 999078, People’s Republic of China; ^g^Department of Earth and Planetary Sciences, University of California, Davis, CA 95616; ^h^Key Laboratory of Cenozoic Geology and Environment, Institute of Geology and Geophysics, Chinese Academy of Sciences, Beijing 100029, People’s Republic of China; ^i^Department of Geological Sciences and Geological Engineering, Queen’s University, Kingston, ON K7L 3N6, Canada

**Keywords:** Hadean, zircon, machine learning, S-type granite, subduction

## Abstract

Due to the scarcity of rock samples, the Hadean Era predating 4 billion years ago (Ga) poses challenges in understanding geological processes like subaerial weathering and plate tectonics that are critical for the evolution of life. The Jack Hills zircon from Western Australia, the primary Hadean samples available, offer valuable insights into magma sources and tectonic genesis through trace element signatures. However, a consensus on these signatures has not been reached. To address this, we developed a machine learning classifier capable of deciphering the geochemical fingerprints of zircon. This allowed us to identify the oldest detrital zircon originating from sedimentary-derived “S-type” granites. Our results indicate the presence of S-type granites as early as 4.24 Ga, persisting throughout the Hadean into the Archean. Examining global detrital zircon across Earth’s history reveals consistent supercontinent-like cycles from the present back to the Hadean. These findings suggest that a significant amount of Hadean continental crust was exposed, weathered into sediments, and incorporated into the magma sources of Jack Hills zircon. Only the early operation of both subaerial weathering and plate subduction can account for the prevalence of S-type granites we observe. Additionally, the periodic evolution of S-type granite proportions implies that subduction-driven tectonic cycles were active during the Hadean, at least around 4.2 Ga. The evidence thus points toward an early Earth resembling the modern Earth in terms of active tectonics and habitable surface conditions. This suggests the potential for life to originate in environments like warm ponds rather than extreme hydrothermal settings.

## Significance

The source of Earth’s oldest materials has been debated using traditional approaches to trace elements and isotopes of zircon. Using a single geochemical proxy, like P contents or oxygen isotopes, for source fingerprinting can be ambiguous. Here, we employ machine learning in multidimensional space, incorporating an array of zircon trace elements. This approach is beneficial for low-P zircon, prevalent in the Hadean. We find significant proportions of S-type granite in the Hadean Jack Hills zircon as far back as 4.24 Ga. These proportions exhibit regular variations in supercontinent-like cycles, mirroring patterns observed in global detrital zircon throughout Earth’s history. This suggests that exposed continents, weathering, and subduction-driven plate tectonics were active since the Hadean, facilitating the habitability of early Earth.

The estimated timing of the onset of subduction, spanning from the Hadean to Neoproterozoic, has been debated for decades (1–5). The crucial question is whether plate tectonics existed before or after 4 billion years ago (Ga), with particular emphasis on the Hadean Eon, which holds profound significance for understanding the geodynamic processes of early Earth (6). Notably, intensive debate over the tectonic regime on early Earth has focused on two widely discussed hypotheses, stagnant-lid tectonics (4, 7–11) and modern-like plate tectonics (6, 12, 13), about which contentious observations of Hadean zircon still persist. For example, the Hf isotope compositions of Hadean zircon (9) have been interpreted as evidence of a stagnant-lid regime and the absence of plate-tectonics-driven cycling. In contrast, Hf isotope cycling (6) and low heat flow inferred from inclusion-bearing zircon (13) argue for a subduction environment similar to modern plate tectonics. To address these discrepancies, it is necessary to determine the provenance of Hadean zircon and further determine the congruency (or incongruency) of these earliest tectono-magmatic features with tectonic evolution throughout geological history.

The absence of a rock record on Earth before 4.02 Ga (14) poses a great challenge to searching for robust evidence of the presence or initiation of plate movements even earlier in the Hadean Eon (>4.0 Ga). The Jack Hills zircon, with ages up to 4.4 Ga (15) and representing 95% of Hadean zircon, is thus the key to address the tectonic regime on early Earth. By searching for the first occurrence of “S-type” detrital zircon sourced from sediment-derived (S-type) granite, closely related with continental collision, orogeny, and crustal thickening (16–20), critical insights into the operation of plate subduction on early Earth could be gained. However, previous work indicates equivocal results concerning the existence of ancient S-type detrital zircon. A minor fraction of detrital zircon with primary muscovite inclusions (21), elevated δ^18^O values (22), or high aluminum (Al) contents (23, 24) have been used to argue for the evidence of S-type zircon as early as the Hadean. However, muscovite inclusions in zircon can also occur in mafic and felsic rocks, as found in the Bushveld Complex (25). Also, the peraluminous threshold based on the Lachlan Fold Belt S-type zircon (Al >4 ppm) likely underestimated the proportion of peraluminous JH zircon (about <10% for pre-3.8 Ga zircon), due to the majority of JH zircon bearing lower Al content than the Lachan I-type (24). Nonetheless, trace element characteristics in detrital zircon (e.g., phosphorus [P] contents and related trace element discriminant diagrams) have been used to argue for the predominance of I-type zircon sourced from igneous (I-type) granite during the Hadean and the whole succeeding Archean Eon (4.0 to 2.5 Ga) (20, 26). Therefore, strong debate over the provenance of Hadean zircon persists, further limiting an understanding of the tectonics of early Earth.

**Fig. 1.**
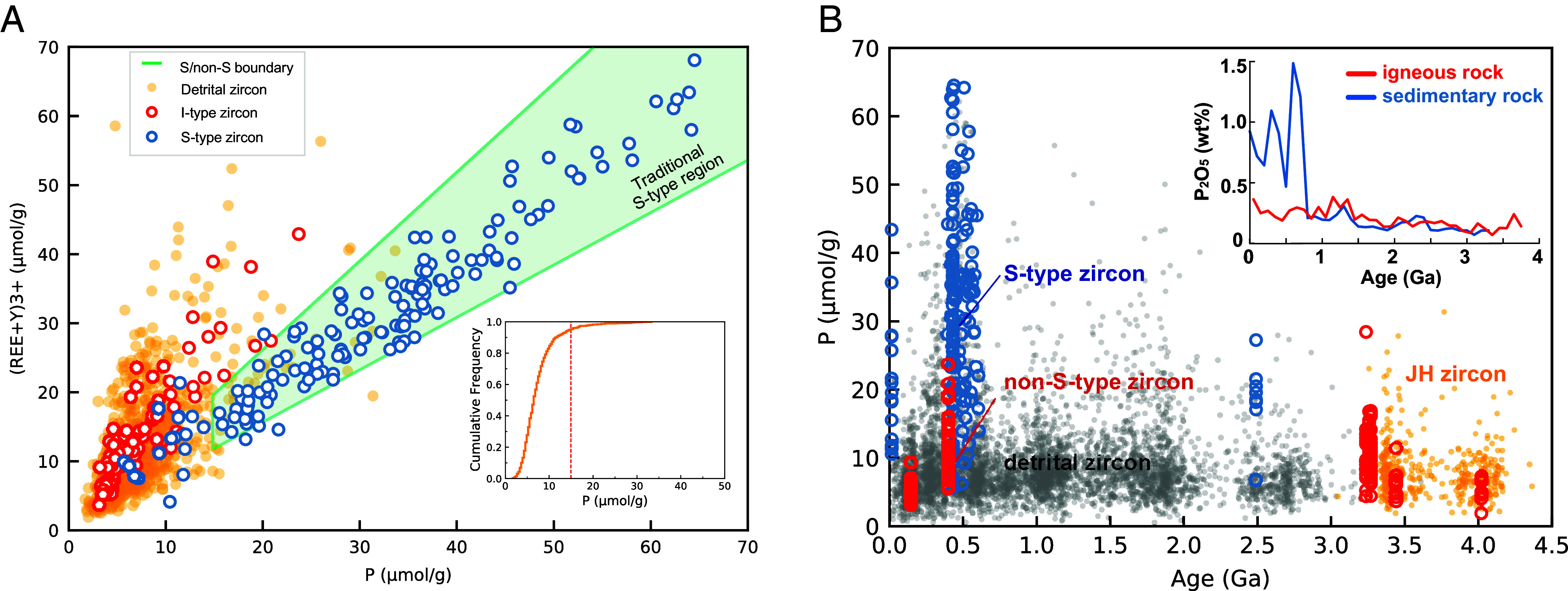
Traditional discriminant diagram of non-S-type (including TTG) and S-type zircon. (*A*) Diagram showing the boundary of I-(TTG) and S-type zircon and the trace element distribution of detrital, I-(TTG) and S-type zircon. Green shadow, upper line and lower line show the “traditional S-type region” and related I/S boundary (20), that zircon with *P* > 15 (μmol/g) and 0.77*P < (REE+Y) < 1.23*P is S-type zircon, and otherwise is non-S-type zircon. The inset shows the cumulative frequency of all Hadean–Archean detrital zircon. (*B*) P contents of detrital zircon and I- and S-type zircon used in this study (Dataset S1). The *Inset* shows P-content time series for igneous and sedimentary rocks from (33, 34) as bootstrapped averages in 10-million-year bins.

Provided that the trace elements in zircon are interconnected and organized by the lattice strain model (27, 28) and the charge balance rule (29–31), the trace element distribution of zircon has the potential to be a more reliable fingerprint for the source of zircon. However, all previously utilized two-dimensional element-element diagrams, demonstrating a significant area of overlap for I-/S-type zircon (*SI Appendix*, Fig. S2), are thus unable to clearly distinguish S-type zircon. Likewise, other traditional combined trace element indexes or diagrams have pitfalls in recognizing S-type zircon. For example, although REE+Y vs. P diagrams can correctly recognize 100% of I-type (including tonalite–trondhjemite–granodiorite [TTG]) zircon, there is a tradeoff as the accuracy for recognizing S-type zircon is only 84% (Figs. 1*A* and 3*A*). Also, according to the confined S-type region (Fig. 1; as defined here with the traditional P criterion), the recognition accuracy for low-P (P ≤ 15 μmol/g) S-type zircon is 0% using a traditional REE+Y vs. P diagram (Fig. 3*A*). This shortcoming, indicating that all zircon with low P characteristics will be considered as I-type zircon, renders this method unable to recognize low-P S-type zircon. Notably, low-P (<15 μmol/g) zircon account for more than 95% of all Hadean–Archean detrital zircon (Fig. 1*A*). In the Phanerozoic, S-type zircon do indeed display the characteristics of elevated P contents, as a result of being sourced from sedimentary rocks bearing much higher P contents than those of igneous rocks (32). However, such a distinct difference in P contents between sedimentary and igneous rocks does not persist throughout Earth history. For the critical interval in question from the Hadean to the Paleoarchean (>3.2 Ga), when the average P contents of sedimentary rocks are comparable to those of igneous rocks (Fig. 1 *B*, *Inset*), rendering Hadean and Paleoarchean S-type zircon, if they existed, essentially identical to low-P I-type zircon in terms of P content alone (32). Thus, when considering the origin of Hadean–Archean zircon, the core problem to solve is how to recognize low-P S-type zircon.

To provide robust constraints on the sources of Hadean–Archean zircon, a tool that takes full advantage of the overall trace element characteristics of zircon to identify S-type zircon with high accuracy of classification is needed, especially for low-P S-type zircon. New machine learning approaches reported in recent two studies were recently applied to attempt such holistic classification, but several apparent pitfalls are to be avoided. One S-/I-type/TTG zircon machine learning classifier reported (35) is problematic on behalf of colinearly related features used in the model, incorrect use of data normalization for the prediction set, and the overfitting problem (*SI Appendix*). Another machine learning approach to S-type zircon classification (36), in which too many elements (17 elements involved) were used in the training of the S-/I-type zircon classifier, leads to an inadequate amount of available detrital zircon data due to the lack of zircon with the complete (large) set of required trace element data. Furthermore, neither study focused on the classification of low P S-type zircons. Learning from these pioneering efforts, we develop here a machine learning classifier to recognize S-type zircon from non-S-type zircon, based on nine trace elements specifically selected and filtered, including P, Y, Ce, Sm, Eu, Dy, Lu, Th, and U. By employing this nine-dimensional machine learning classifier, we are capable of confidently tracing the sources of the Hadean Jack Hills zircon and further reveal the evolution of detrital zircon for the whole Earth throughout geological history.

## Preparing a S-Type vs. Non-S-Type Zircon Dataset for Machine Learning

In machine learning, one must first most critically decide which data to include in the training and test sets in order for the classification of the prediction set (the unknowns) to be meaningful. To construct a robust classifier to recognize S-type zircon, it is necessary to fully exploit the hidden multidimensional information coded in the trace element fingerprints of zircon. Thus, we apply machine learning classification algorithms, which are able to deal with multiple geochemical input data to accomplish this task. We mitigate the “open set” classification issue (i.e., the shoehorning of unknowns into a prescribed number of finite classifications) by posing classification as a simple either/or question: either sediment was involved in the formation of a granite (S-type zircon) or sediment was not involved (non-S-type zircon). Thus, our aim is to build a classifier of S-type vs. non-S-type zircon. Nevertheless, an unavoidable question is what types of zircon should be included in the non-S-type zircon training set. A Hierarchical Clustering tree demonstrates that the dataset used—composed of all zircon in the training, test, and prediction sets—manifests exactly two clusters as the most appropriate groups, no matter for Phanerozoic or Hadean zircon (*SI Appendix*, Fig. S4). Principal component analysis (PCA) and t-distributed Stochastic Neighbor Embedding (t-SNE) (*Materials and Methods*) both show that there are definitely no other types (e.g., A-type zircon or M-type zircon) apart from S-type zircon, I-type zircon, and TTG zircon in the prediction set (*SI Appendix*, Fig. S5). Therefore, we resolve that including I-type and TTG zircon in the training/test sets is sufficient for compiling non-S-type zircon. A complete workflow for our machine learning models is provided in *SI Appendix*.

First, the raw dataset prepared for machine learning includes zircon trace element compositions of the Jack Hills zircon, and non-S- and S-type zircon collected from various locations globally and throughout geological history (*SI Appendix*, Fig. S1). Typical S-type zircon from granites from i) the Lachlan Fold Belt, Australia, ii) Lusatian, Germany, and iii) S-type zircon from Himalaya leucogranite, and iv) the Western Block of North China Craton (20, 26, 37). Non-S-type zircon were compiled in this study, from i) the Lachlan Fold Belt, Australia, ii) I-type zircon from granodiorites of the Yangtze River Metallogenic Belt, eastern China (ca. 140 Ma) (38), iii) the Acasta Gneiss Complex, Canada, iv) TTG zircon from the Badplaas pluton (tonalite, ca. 3,270 million years ago [Ma]) and the Stentor pluton (biotite trondhjemite, ca. 3,258 Ma), Barberton granite–greenstone terrane, South Africa (39). Lachlan Fold Belt I- and S-type zircon and S-type zircon from Himalaya leucogranite (near Cuona leucogranite) without reported ages are estimated at 400 Ma, 430 Ma, and 15 Ma, respectively (40, 41). Zircon with abnormally high Th (>1,000 ppm) and/or U (>3,000 ppm) contents, which are around one order of magnitude higher than median contents (42), and potential metamorphic zircon (43), are excluded. Only zircon with complete input features, primary magmatic signatures, and concordant ages (<10% discordance) are included in our dataset. Both the clean zircon criterion (La < 0.1 ppm) (44) and the primary composition filter (37) were applied to select zircon. Despite the challenge of the limited number of qualified zircon available (e.g., in the GEOROC database, only 0.4% of zircon, regardless of their sources, qualify our criteria), we nonetheless compile an exhaustive and adequate zircon geochemical dataset for machine learning. The dataset consists of 1,345 zircon, including 154 S-type zircon, 220 non-S-type zircon (TTG zircon and I-type zircon), 971 Hadean–Archean detrital zircon (403 of which are Jack Hills zircon) (Dataset S2), and a global compilation of 4,759 detrital zircon ranging from 3,600 to 14 Ma (20) (Dataset S3).

Second, we split our dataset into training, testing, and prediction datasets. All known 374 zircon, containing well-classified I- and S-type zircon from granites and TTG zircon ranging from 4.02 to 0.02 Ga from North America, Europe, Africa, and Asia, are randomly separated into two groups: 80% as the training set and the remaining 20% as the test set. The training dataset, including 123 S-type zircon and 176 non-S-type zircon, is not large but adequate for training the machine learning models, as evidenced by the learning curve on experiments incrementally increasing the number of training set data used to train the machine learning models (*SI Appendix*, Fig. S6). The test set, composed of 31 S-type zircon and 44 non-S-type zircon, is used to statistically evaluate the classification performance of the trained machine learning model. Finally, the 971 Hadean–Archean detrital zircon with unknown origin (including the Jack Hills zircon) are designated as the prediction set, whose magmatic source is determined after establishing that the machine learning model could accurately distinguish non-S- and S-type zircon in both the training and test sets.

**Fig. 2.**
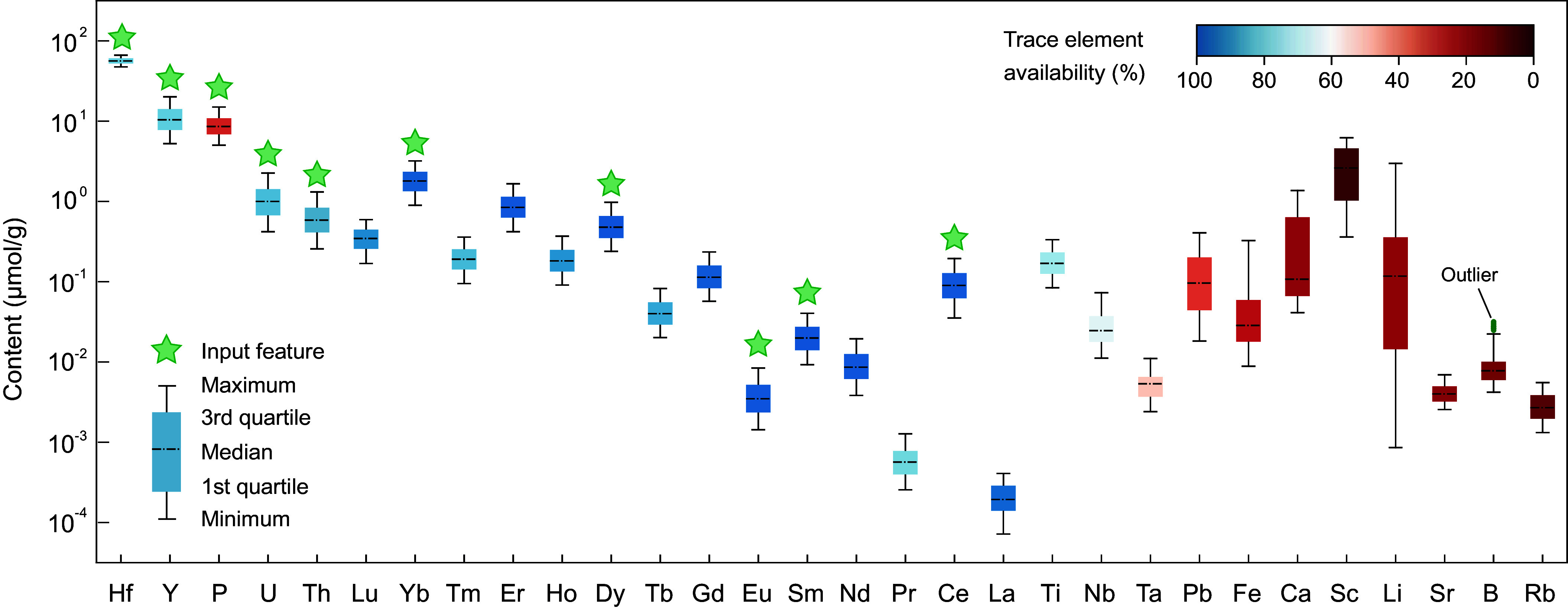
Trace element fingerprints of zircon. These bars show the composition and availability of the top 30 most common trace elements in the zircon dataset (*SI Appendix*, Table S1) and a detrital zircon dataset from ref. 22 (Dataset S2). Box-and-whiskers plot shows the maximum, 3rd quartile, median, 1st quartile, and minimum values. The highest and lowest 20% of values of each element are excluded to reduce scatter. Trace element availability is calculated by *n*/*N*, where *n* is the available trace element data and *N* is the size of the dataset. Those elements used as input features for machine learning are indicated with stars.

Third, we selected input features. According to the trace element fingerprint of zircon (Fig. 2 and *SI Appendix*, Table S1), 9 trace elements were selected as input features for the machine learning model, including Y, P, Th, and U, and typical light, middle, heavy, and redox-sensitive REE (Sm, Dy, Yb, Ce, and Eu), where Th and U are decay-corrected to reflect their original contents when the zircon formed. Three rules are used in selecting input features. The first rule is data availability. To create a large robust dataset, an input feature must be widely available for many grains. Y, Th, U, and REE (except for La and Pr) are the most available trace element data, with >80% zircon in our compiled dataset reporting them (Fig. 2 and Dataset S2). Second is data importance. Y and P are most abundant in zircon and collectively account for >85 mol% of trace element concentrations in the lattice of zircon. Th, U, and REE, although less abundant, almost exclusively occupy the remaining site in the lattice of zircon. Third is data quality. The “clean zircon” filters can control for the data quality of the input features selected by mitigating the influences of alteration and contamination from most mineral inclusions, especially REE carriers (e.g., monazite, apatite, and xenotime). Therefore, for Nb, Ta, Al, Ti, and Ca, all of these are below data quality control standard, and other elements with scarce data availability (<15%; *SI Appendix*, Table S1) are not selected as input features. Our input feature selection also sought to remove colinear elements and features with low importance based on the machine learning techniques in this article (*Materials and Methods* and *SI Appendix*, Fig. S7). We eventually arrive at the available and preferred input features including the most abundant trace elements, P and Y (which account for ~90% of total trace element concentrations), typical LREE Sm, MREE Dy, HREE Lu, and several redox-sensitive trace elements (Ce, Eu, Th, and U) in Phanerozoic I-type zircon, Archean TTG zircon, and Phanerozoic S-type zircon. These trace element fingerprints are further processed using normalization method (*Materials and Methods*) to transform raw trace element data into normal distributions with zero mean and unit variance (45) to make the dataset conducive to machine learning.

## A Machine Learning Classifier for S-Type Zircon

We experimented with 11 mainstream machine learning classification algorithms (46–56), including Logistic Regression (LR), Decision Tree (DT), Multilayer Perceptron (MLP), Voting, Bagging, k-Nearest Neighbor (KNN), AdaBoost, Gaussian Naive Bayes (GNB), Bernoulli Naive Bayes (BNB), Support Vector Machine (SVM), and Transductive Support Vector Machine (TSVM) (more details provided in *Materials and Methods*). We employed the F1-score to assess the effectiveness of the models (*SI Appendix*). Upon comparing the performance of various algorithms (*Materials and Methods* and *SI Appendix*, Table S2), we found that TSVM had the highest F1-score (0.96), with LR (0.96), KNN (0.95), and MLP (0.95) trailing closely behind. DT, SVM, Bagging, Voting, and AdaBoost also displayed high accuracy rates >0.90, while GNB and BNB produced lower accuracy rates with 0.86. Therefore, TSVM, KNN, LR, and MLP were chosen as our main classifiers to detect S-type vs. non-S-type zircon. Furthermore, our primary focus lies in classifying low-P zircon (*P* < 15 mmol/g), which comprise the majority of Hadean detrital zircon; thus, the F1 score of low-P zircon must be considered for the final selection of machine learning models. According to the F1 score of each algorithm specifically for low-P zircon, TSVM produced the highest F1 score (0.95), followed by LR (0.95), MLP (0.93), and KNN (0.93) (*SI Appendix*, Table S2). Based on these performance evaluations, TSVM, for which a schematic diagram of the model training principle is shown in *SI Appendix*, Fig. S3, was determined as primary classifier for identifying low-P zircon.

**Fig. 3.**
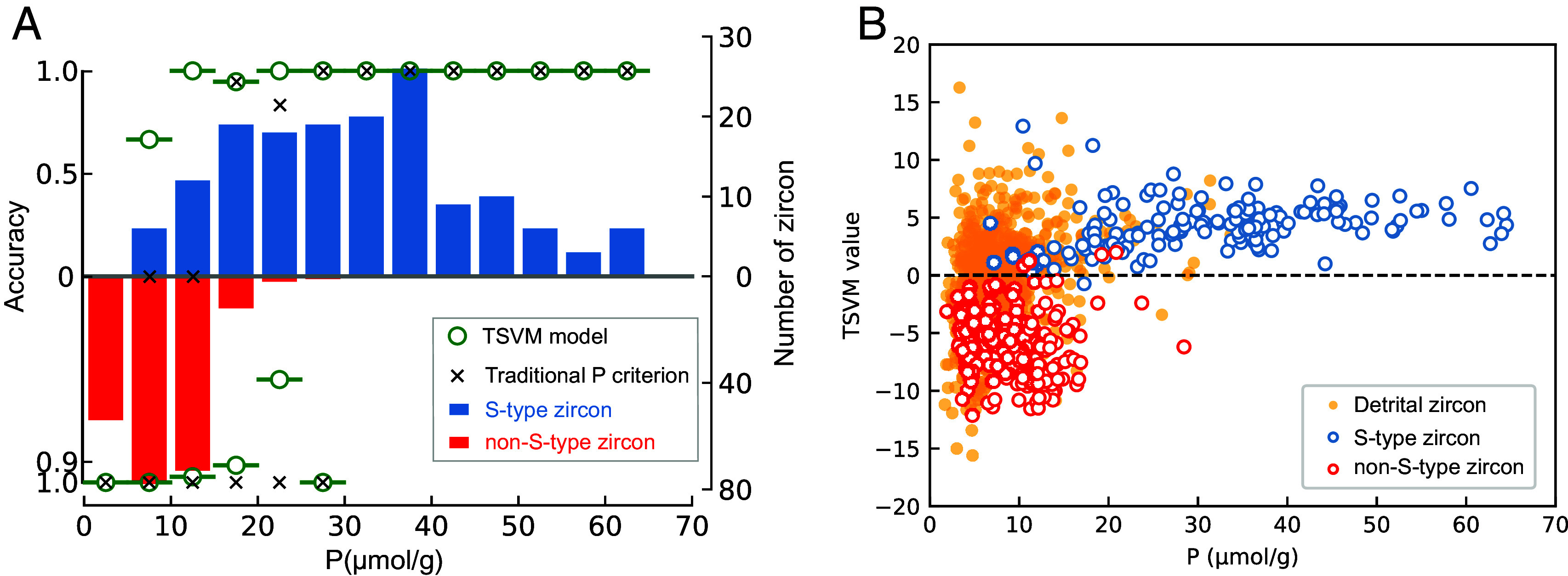
Comparison of the machine-learning method (TSVM model) and the traditional P criterion in recognizing S-type zircon. (*A*) The accuracy of both methods for non-S- and S-type zircon with different P contents in the dataset. The distribution of P contents for non-S- and S-type zircon are shown in the red and blue histograms, respectively. The accuracy of each model in our dataset (both training and test sets) is calculated using a bin size of 5 μmol/g (Dataset S2). (*B*) Clear-cut discrimination of non-S- and S-type zircon by TSVM value = 0, where TSVM values mean the prediction output value of TSVM (defined in *Materials and Methods*). Blue open circles are S-type zircon, red open circles are non-S-type zircon, and orange filled circles are detrital zircon with Hadean to Archean ages. TSVM value is calculated based on the decision function of the machine-learning model (*Materials and Methods*), TSVM value = (5.66) * P + (0.54) * Y + (–1.66) * Ce+ (0.49) * Sm+ (–1.47) * Eu+ (–0.69) * Dy+ (–3.90) * Lu+ (0.42) * Th+ (0.58) * U, where >0 means S-type zircon and <0 indicates non-S-type zircon.

Through this classifier combination, we established an extensive and precise technique for identifying Hadean zircon, particularly well-suited for those with low-P compositions. Using our machine learning method TSVM, we have achieved cutting-edge accuracy in identifying S-type zircon, with a misclassification rate of <5% for S-type zircon that are classified as non-S-type (i.e., false negatives) and 0% for non-S-type zircon that are identified as S-type (i.e., false positives; Fig. 3*A*). Especially for S-type zircon with *P* ≤ 15 mmol/g, the recognition accuracy has been greatly enhanced from 0% for traditional P criterion to >85% for TSVM. This considerable boost is of great aid in the identification of Hadean detrital zircon, featured by low P concentrations. The reliability of the prediction of Hadean zircon is also further guaranteed during its performance on the test set which includes Phanerozoic and Archean zircon, thus proving its insensitivity to the ages of the zircon, in addition to being insensitive to zircon P contents and insensitive to geographic location. Thus, the classifier is applicable to all detrital zircon globally and throughout geological history.

Aside from the considerable improvement of prediction performance on the test set, the effectiveness of our machine learning model can be further evidenced by the mathematical function of the determined hyperplane, which is correspondingly consistent with the specific geochemical characteristics of S-type zircon (*Materials and Methods*). The spider diagram (*SI Appendix*, Fig. S9) demonstrates that the REE pattern of the predicted Hadean S-type zircon is comparable to Phanerozoic S-type zircon, which in turn proves that our machine learning model has learned the intrinsic REE pattern and element features, regardless of age and geological provenance.

## Discussion

### Origin of Hadean Zircon.

With demonstrated performance of robust recognition of zircon types, we argue that our machine-learning model can provide the most unequivocal determination of the magmatic origins of ancient detrital zircon. The result obtained by our method reveals that S-type zircon are found in 12 tested locations of Hadean–Archean detrital zircon (*SI Appendix*, Table S3). Strikingly, the Hadean Jack Hills zircon that represent >95% of all Hadean zircon are composed of 35% S-type zircon. Such high proportions of S-type zircon or even higher are also found from four locations with Archean detrital zircon (>50%; *SI Appendix*, Table S3). We also employed TSVM to predict Hadean clean zircon from the recently discovered Green Sandstone Bed in South Africa (57, 58) (Dataset S3), which yields a similarly high proportion of S-type zircon (39%), quite consistent with the Jack Hills result (35%) (*SI Appendix*, Fig. S13). These consistent results from different locations suggest that such high proportion of S-type zircon in the Hadean and early Archean is likely to be globally prevalent. We provide a note of caution concerning the prediction of Hadean zircon based on younger zircon, particularly in light of compositional variations of magma throughout Earth’s history. As Hadean magma is characterized by lower Zr (i.e., likely delaying zircon saturation) (57, 59) and higher U content, we demonstrate that such an effect has a negligible impact on the overall trend of global S-type zircon proportion (*SI Appendix*). Additionally, to a certain extent, the high accuracy of recognizing S-type zircon for Archean–Phanerozoic zircon in the test set also suggests that the classification is not significantly skewed by magmatic compositional shifts over time. Finally, our sensitivity test using three alternative machine learning models reveals similarly high and fluctuating proportions of Hadean S-type zircon (*SI Appendix*, Fig. S8).

**Fig. 4.**
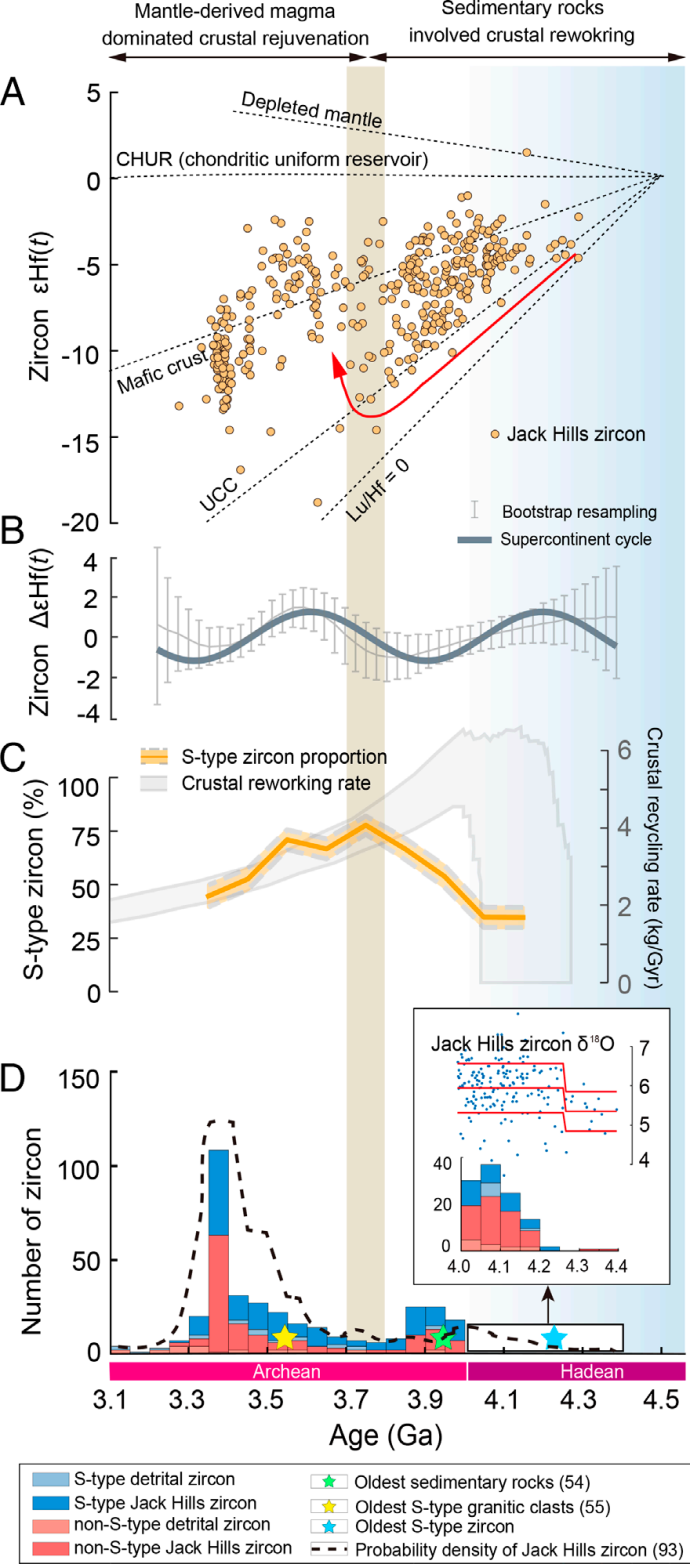
S-type detrital zircon on early Earth and the evolution of magma sources. (*A*) Hf isotopes of the Jack Hills zircon (9, 62, 63). The isotope trajectories of the putative depleted mantle (64), mafic crust (65), and upper continental crust (UCC) (66) are shown for reference, assuming silicate Earth differentiation at 4.5 Ga. Arrows mark the trend of prolonged internal crustal recycling of Hadean to Eoarchean crust and the transition at 3.8 to 3.7 Ga toward increasing juvenile contributions to magma sources. (*B*) Detrended Hf isotope of Hadean–Archean detrital zircon (67). (*C*) Bootstrapped average of S-type zircon proportion through time with 1 SE (*SI Appendix*, Table S4) and crustal recycling rate (68). (*D*) Stacked histogram of the distribution of I- and S-type zircon through time as classified by machine learning, including detrital zircon from the Jack Hills, Australia, and 12 other locations. The *Inset* shows δ^18^O step-change of Jack Hills zircon (61) and a histogram of zircon older than 4.0 Ga. The dashed line shows the probability density plot of detrital zircon along the traverse through the Jack Hills belt (69). See *SI Appendix*, Table S3, for the individual distributions of S-type zircon in each of the 12 locations studied. All the zircon presented have U-Pb ages that are <10% discordant.

These results starkly contrast with previously conflicting studies using traditional methods indicating only rare, or almost no ancient S-type zircon (21, 26). Although highly evolved I-type magmas may have weakly peraluminous compositions resembling S-type magma in a late stage of magmatic evolution (60), such late-stage I-type zircon are negligible in number (23) with trace element compositions similar to I-type rather than S-type zircon (24). The risk of overestimating the proportion of S-type zircon by misidentifying such uniquely evolved I-type zircon as S-type zircon (i.e., false positives) is very limited, and can thus not explain such a widespread and abundant proportion of S-type detrital zircon with Hadean–Archean ages. In addition, we note that the occurrence of S-type zircon in the Hadean based on our results coincides with a step change of Jack Hills zircons δ^18^O at ca. 4.25 Ga (61) (Fig. 4 *D*, *Inset*). Elevated δ^18^O would be expected if the proportion of S-type zircon increases.

The ubiquitous existence of S-type zircon among ancient detrital zircon provides compelling evidence that a considerable amount of S-type granite—as well as sedimentary rocks as inputs into their magmatic systems—already existed on Earth by as early as at least 4.2 Ga and persisted throughout the rest of the Hadean and the whole Archean (Fig. 4 *C* and *D*). Although the relative proportion of S-type Jack Hills zircon among the Jack Hills zircon population may not be precisely equal to the proportion of S-type granite among their source granite(s) (70), it still suggests the potentially ubiquitous existence of S-type granite during the Hadean to early Archean. Further, the result pushes the existence of both the oldest sedimentary rocks at 3.95 Ga (71) and the earliest S-type granite at 3.55 Ga (72) 290 and 690 million years earlier than previously known, respectively. An early, dominant, and persistent presence of S-type granite and sedimentary rocks strongly suggests that Earth already had a considerable amount of crust exposed above sea level by at least 4.2 Ga. This age indeed provides a minimum age for these geological processes being fully operational on Earth as such a product as S-type granite requires an extensive preprocessing sequence including the weathering of crust, the deposition of sedimentary rocks, and their burial and incorporation into deep magmatic systems. Regardless of the possible locations and tectonic settings of this early and long-lived emerged crust, such as seamounts from mantle plumes (73), magmatic arcs from plate tectonic subduction (74), and/or uplifted crater rims from impacts (75), with the participation of an active hydrosphere, it was effectively weathered, eroded, and deposited to form sedimentary rocks, and then reworked by some burial mechanism(s) in order to enter magma chambers.

Archean–Haden S-type zircon is distinct for their low-P contents and, correspondingly, also their low REE (*SI Appendix*, Fig. S10 *C* and *D*). These features are likely due to the geochemical environment in which these zircon were generated, that is, they likely are reflective of early low-P sediments due to the preliminary stage of Earth’s development (76). Modern S-type zircon are crystallized during magmatic evolution with gradually decreasing P content caused by the crystallization of apatite (33). Similarly, according to our observed trends in P and REE+Y (*SI Appendix*, Fig. S10*B*), such decreases accompanying increasing fractional crystallization with apatite-out is also an important factor constraining P and REE contents for ancient S-type zircon. Additionally, Hadean S-type zircon is characterized by Ce and Eu anomalies, similar REE patterns with young low-P S-type zircon, and lower HREE than non-S-type zircon (*SI Appendix*, Fig. S9). These observations lead us to infer that Hadean zircon share a similar formation mechanism with modern S-type zircon, that is, the negative Eu anomaly is caused by equilibration with a feldspar-rich residuum and the positive Ce anomaly reflects the redox conditions of the source magmas (77). Titanite crystallization may also have an essential bearing on the partitioning of Ce and Eu (78). In summary, the only difference of Jack Hills S-type zircon from modern S-type zircon is the overall low-P feature owing to the low-P source of ancient rocks (34), while other similar REE features hint at a strikingly similar petrogenesis.

Although high P content is seen as a diagnostic characteristic for S-type zircon (26) in previous studies, low-P S-type could also be explained well in terms of petrogenesis. Previous work suggests that rare elements do not have the same partition coefficients for xenotime substitution, with experiments showing LREE/P < 1, MREE/P around 1, and HREE/P > 1 (79). When LREE:HREE are not equivalent to 1:1 in the substitution of REE^3+^ for the site of Zr^4+^, this leads to great variation in REE/P; thus, natural zircon are often not completely distributed along a 1:1 xenotime line (79, 80). Several low-P modern S-type zircons in the training set are distinctly scattered along the trend of I-type zircon (*SI Appendix*, Fig. S10*B*). Both S-type zircon and I-type zircon may have REE/P > 1, so this deviation away from xenotime line for Hadean–Archean zircon cannot be solely attributed to the notion that these zircons are not S-type. The P abundance in low-P magma sources could influence the REE partitioning in zircon by limiting the crystallization of REE-bearing accessory minerals such as apatite and monazite (33, 81), especially. In addition, the presence of interstitial Li^+^, Mg^3+^, Fe^2+^, and Al^3+^ cations (30) or Nb and Ta (82) could also possibly contribute to additional charge balance of trivalent ions REE allowing for lower P contents, making higher REE/P.

### Hadean Subduction-Driven Plate Tectonics.

Our observation is independently corroborated by the Hf isotopes of Hadean Jack Hills zircon that indicate a transition in their magma sources at ca. 3.8 Ga (Fig. 4*A*). Before 3.8 Ga, linearly increasingly unradiogenic Hf signatures suggest the magmatic trend of a largely consistent upper-continental-crust-type reservoir was involved in magma sources (62) (Fig. 4*A*). This trend of sustained crustal reworking before 3.8 Ga is consistent with the formation of S-type granite and can thus account for the high and increasing proportion of S-type Jack Hills zircon from 40 to 80% from 4.2 to 3.8 Ga (Fig. 4*C*). After 3.8 Ga, the increase in ε_Hf(_*_t_*_)_ of the Jack Hills zircon indicates juvenile input (recently extracted from the mantle), and the slope of this trend suggests Hadean mafic materials in magma sources (62) (Fig. 4*A*). This wavelength of this supercontinent-like-cycle transition also illustrated by Δε_Hf(_*_t_*_)_ (Fig. 4*B*) from continental crustal reworking to mafic magmatism (67) suggests the operation of early orogenic processes (20). This shift toward radiogenic ε_Hf(_*_t_*_)_ values can account for the increasing amount of non-S-type zircon (I-type zircon or TTG zircon) (Fig. 4*D*) and the decreasing proportion of S-type Jack Hills zircon that reaches as low as 40% by ca. 3.4 Ga (Fig. 4*C*).

**Fig. 5.**
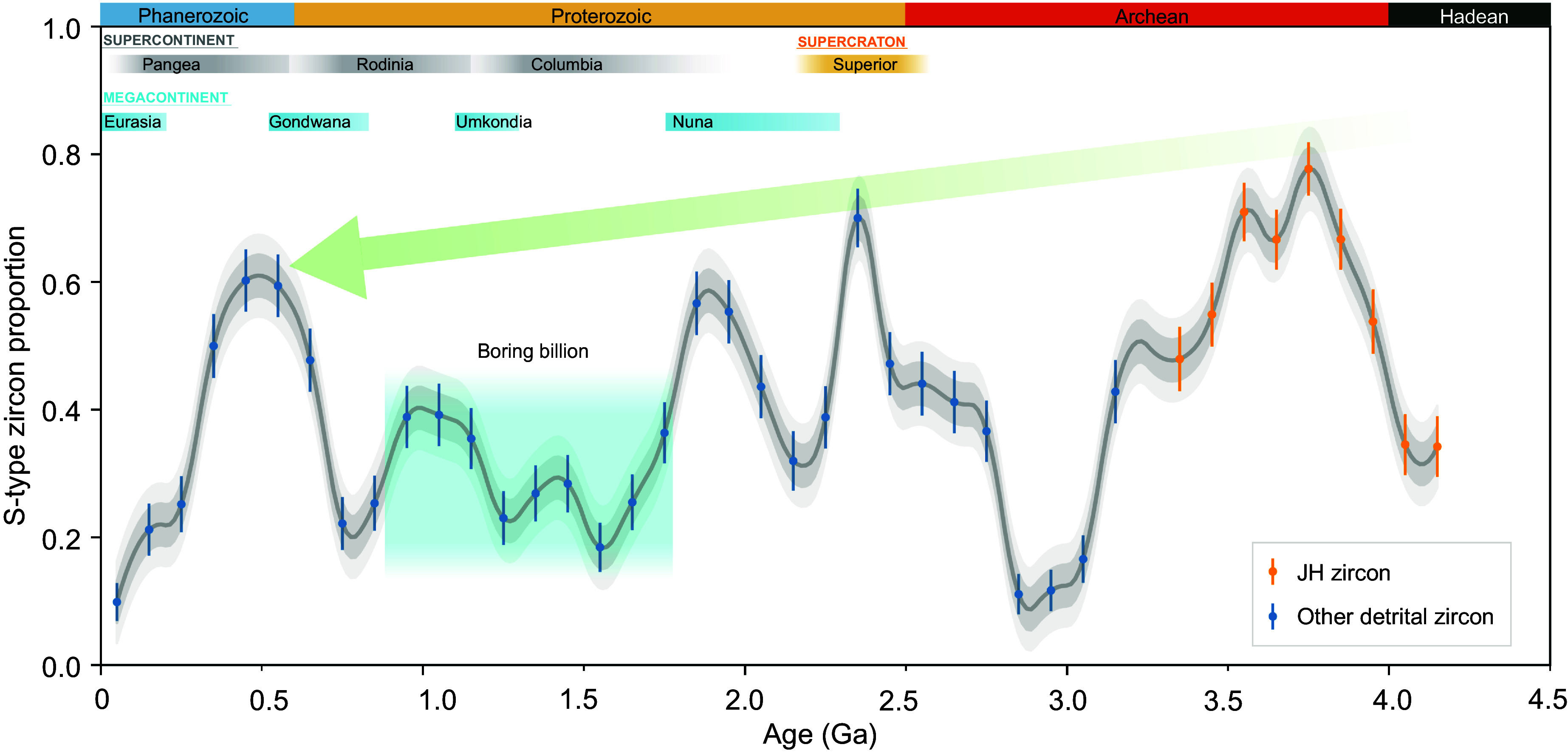
Secular variation of the proportion of S-type detrital zircon throughout Earth history. The proportion of S-type zircon shown as grey curve is calculated by bootstrap method, involving resampling 100 times to calculate the S-type proportion within a bin size of 100 My, as error bars denote ±2 SEM. Jack Hill zircon is shown in orange and other detrital zircon is shown in dark blue. The green trend line shows the peaks decline from Hadean to Phanerozoic. The blue square shadow indicates the “Boring Billion” [a.k.a. “Balanced Billion” (84) ranging from 1.8 to 0.9 Ga. Upper bars indicate supercontinent (gray) and megacontinent (blue) time periods (83). See *SI Appendix*, Table S4, for the calculated proportion of S-type zircon from Hadean to the present day. All the zircon presented have concordant ages (<10% discordance).

S-type granites represented the majority proportion—exceeding 50%—of Hadean granites. The findings revealed by our machine learning model further demonstrate for detrital zircon globally the occurrences of such large proportions of S-type granites repeat throughout geological history. Consistent peaks and valleys in S-type zircon proportion occur throughout time when applying TSVM to predict global detrital zircon. These peaks, surpassing 50% at 0.5, 1.0, 1.8, and 2.4 Ga, interestingly align with the periods of supercontinent assembly, specifically “megacontinents” (Fig. 5), viewed as an early assembly phase of continents before final supercontinent amalgamation (83). Each climbing trend from a lower frequency S-type zircon period to a following higher frequency period corresponds to each of the megacontinents since 2.5 Ga. Documenting that such S-type variations in younger times are clearly associated with megacontinent formation, or more commonly known supercontinent cycles, reinforces a plate tectonic interpretation of the Jack Hills zircon S-type and ε_Hf(_*_t_*_)_ data. Other ML methods (MLP and LR) yielding F1 scores > 0.93 produce quite similar patterns (*SI Appendix*, Fig. S11), supporting the observation of supercontinent cycles in S-type zircon as robust. We thus conclude that subduction-driven plate tectonics has operated on the Earth since the Hadean.

What is more, these S-type zircon proportion cycles show an overall secular decreasing trend (Fig. 5). Higher mantle potential temperatures in the Hadean–Archean would cause more frequent slab breakoff during subduction (85), evidenced by faster crustal recycling rates (68) (Fig. 4*C*), leading to more subduction-related rocks like sediments in subduction channels entering magma chambers and thus yielding a higher proportion of S-type zircon. It has been suggested theoretically that rapid plate motion and ocean formation during the Hadean could be possible with a hydrated heterogeneous mantle (86). It also follows that relatively low peaks in this long-term variation occur during the period known as the Boring Billion (c. 1.9 to 0.9 Ga), a.k.a. Balanced Billion (84), implying relatively subdued plate tectonic and subduction activity. Consequently, it appears that the elevated presence of S-type zircon during the Hadean Eon signifies the existence of early plate tectonic activities akin to those observed in the modern Earth’s geological system, predominantly influenced by plate subduction rather than the recently reported stagnant-lid regime (7).

By establishing systematic machine learning methods to recognize S-type zircon, our research provides valuable insights into not only the plate tectonic mode that operated during the Hadean Eon but also a similarly analogous habitat on the early Earth. Such an early ocean–land configuration allowing for subaerial weathering (87) would have been capable of regulating Earth’s surface temperature and providing bioessential nutrients—both critical requirements for most forms of life on Earth. Thus, our results support recent challenges (88) to the canonical model of the tree of life having bacterial roots in severe hydrothermal environments (89), as the amenable surface conditions depicted here permit the recent revival of Darwin’s “warm little pond” as a competing viable model (73). These early temperate surface conditions resembling the modern Earth thus portray a habitable planet permitting the origin of life at ~300 million years or less after Earth formation.

## Materials and Methods

### Pretraining Selection of Elements for the Machine Learning Model.

Before training, we first calculated the chi-square values of 17 elements for non-S- and S-type zircon to select important elements. It is well known that the classic chi-square test is designed to test the correlation between categorical variables, with a higher chi-square value corresponding to a greater importance for classification. We get the following input feature list of elements sorted by classification importance: P, Y, Yb, Er, Hf, Dy, Th, Ho, Lu, Tm, Ce, Gd, Tb, Sm, Eu, U, and Nd. Then, according to relative importance, we used the Forward Feature Selection method (90, 91), which in our case adds one element each time in order into the input feature list used to train the machine learning model. According to the Forward Feature Selection method, including P, the accuracy of the machine learning model is 95%, whereas excluding P results in an accuracy of <60%. REE elements are also essential and increase the accuracy of the machine learning model for low-P S-type zircon from 0% to 80%. Using Feature Selection Engineering, we selected nine elements as input features that could train the machine learning model with high accuracy afterward.

### Data Normalization.

We used the data normalization method by applying the centered log ratio (CLR) transformation and Feature Scaling method. To avoid the deleterious effect of large disparity between different elements in machine learning classification, we applied the CLR transformation (92) to process our dataset, which normalizes the elements of a sample by the geometric average and logarithmic transformation. Then, the transformed zircon data were further processed using the Feature Scaling method (93) to normalize each element data of all samples to transform raw trace element data into normal distributions with zero mean and unit variance, making the dataset compatible with machine learning. Additionally, we made sure to apply the same scaling statistics to normalize both the testing set and the prediction set, ensuring consistency for the whole dataset.

### Nonoverfitting.

To ensure that our model is not overfitting, we must evaluate its accuracy on both training and validation sets. One way to prevent overfitting is to split the data into a training set and a validation (test) set. We use leave-one-out cross-validation (94) (full details in *SI Appendix*) to evaluate the accuracy of the model and applied the Grid Search method (95) to adjust the hyperparameters to optimize the model’s performance, considering that our dataset is small. Furthermore, we can plot the learning curve for the best model, which depicts how the model’s training and validation accuracy or error changes as we increase the number of training examples. If the model is overfitting, we would observe a substantial gap between the training and validation accuracy as the number of training examples increases. This learning curve (*SI Appendix*, Fig. S6) is evidence that our model is not overfitting, and it has learned to generalize well on unseen data.

### Comparison of Different Machine Learning Models.

We chose the classical 11 machine learning algorithms to train the classifier for non-S-/S-type zircon. Multilayer Perceptron (MLP) is a neural network architecture with multiple layers, which can capture the nonlinear relationship between the features and the target variable (39). Voting and Bagging are ensemble learning methods that combine multiple classifiers to improve the classification accuracy (53, 54). KNN is a lazy learning algorithm, which classifies the samples based on similarity to their neighbors. AdaBoost is another ensemble learning method which assigns higher weights to the misclassified samples in each iteration (55). Random tree is a decision tree-based algorithm that randomly selects subsets of features and samples to train multiple trees (50). GNB is a probabilistic classifier based on the Bayes theorem with an assumption that the features are independent (47). BNB is a variant of GNB for binary features (48). TSVM is a semisupervised learning algorithm that extends the normal SVM to also incorporate unlabeled data for classification tasks (56). The key idea of TSVM is to exploit the underlying structure of both labeled and unlabeled data by iteratively refining the decision boundary through an interpolation process, achieving better classification performance than normal SVM.

### PCA and t-SNE.

Principal component analysis (PCA) is a dimensionality reduction method to transform high-dimensional data into lower dimension by finding the direction of maximum variance for dataset via calculating maximum eigenvalues and corresponding eigenvectors (96). In this article, PC1 and PC2 diagram for all zircon data containing known and unknown zircon data (*SI Appendix*, Fig. S5*A*), the first two components extracted by PCA, contains 95% feature information in total. And t-distributed Stochastic Neighbor Embedding (t-SNE) is also a method to reduce dimension and visualize clustering (97). It could map all elements of samples to these two dimensions shown in *SI Appendix*, Fig. S5*B*. Both methods show that the prediction set is scattered in the domain of the training set and test set. That means there are no other zircon types (e.g., an unknown type zircon or A-type zircon) in the prediction set.

### Mathematical Function of TSVM Hyperplane.

As shown in Fig. 3*B* and *SI Appendix*, Fig. S3, the TSVM algorithm classifies the zircon by finding a multidimensional plane that best separates the two types of zircon. The function of this hyperplane (shown in Eq. **1**) will give information by the coefficients and intercept of this hyperplane, where Th and U means Th and U content with time decay-related correction here. The coefficient of each element represents its influence on the classification of non-S-/S-type zircon. The greater absolute value of the coefficient represents more influence, and plus or minus sign indicates a positive or negative impact on S-type zircon (the positive samples) and the non-S-type (the negative samples). It explains that the P element is the most important for the classification, followed by Lu, Ce, and Eu. And Ce, Eu, Dy, and Lu affect negatively on the S-type, indicating that lower Ce, Eu, Dy, and Lu may represent S-type zircon characteristics. This observation regarding influence of elements on zircon classification aligns with the characteristics displayed in *SI Appendix*, Fig. S9, which illustrates the distinct REE compositions of non-S-type and S-type zircons. For example, S-type zircon have more negative Eu anomaly and less positive Ce than non-S-type zircon corresponding to the negative coefficient of Ce and Eu.

[1] $$\begin{array}{l} \left(5.66\right)\;*\;P\;+\;\left(0.54\right)\;Y\;+\;\left(-1.66\right)\;*\;\text{Ce}\;+\;\left(0.49\right)\;*\;\\ \text{Sm +}\left(\text{-1.47}\right)\text{* Eu +}\left(\text{-0.69}\right)\;\text{* Dy +}\left(\text{-3.90}\right)\text{*}\\ \text{Lu +}\left(\text{0.42}\right)\text{* Th+}\left(\text{0.58}\right)\text{* U- 1.60}=0. \end{array}$$

More details of our machine learning models and their workflow can be found in *SI Appendix*.

## Supplementary Material

Appendix 01 (PDF)

Dataset S01 (XLSX)

Dataset S02 (XLSX)

Dataset S03 (XLSX)

## Data Availability

All data, code, and materials are available online at https://github.com/jiangjilian/zircon_recognition. The work uses open-source software (Python and Python packages). All machine learning algorithms in this article can be implemented by calling functions from Python library scikit-learn, with more details provided at https://scikit-learn.org/stable/index.html (98, 99).
