# Supplementary material for: Sediment subduction in Hadean revealed by machine learning
Source: Proc Natl Acad Sci U S A. 2024 Jul 8;121(30):e2405160121. doi: 10.1073/pnas.2405160121 (PMC11287277; doi:10.1073/pnas.2405160121)
Supplement: Supplementary file 1 — Appendix 01 (PDF) [file pnas.2405160121.sapp.pdf]

PNAS

Supporting Information for

Sediment subduction in Hadean revealed by machine learning

Jilian Jiang[1,2,†], Xinyu Zou[3,†], Ross N. Mitchell[1,2*], Yigang Zhang[1,4*], Yong Zhao[6], Qing-Zhu Yin[7], Wei Yang[5,2], Xiqiang Zhou[8,2], Hao Wang[1,2], Christopher J. Spencer[9], Xiaocai Shan[1], Shitou Wu[1,2], Guangming Li[3,2], Kezhang Qin[3,2], Xian-Hua Li[1,2]

[1]State Key Laboratory of Lithospheric Evolution, Institute of Geology and Geophysics, Chinese Academy of Sciences, Beijing, 100029, China
[2]College of Earth and Planetary Sciences, University of Chinese Academy of Sciences, Beijing 100049, China
[3]Key Laboratory of Mineral Resources, Institute of Geology and Geophysics, Chinese Academy of Sciences, Beijing 100029, China
[4]Key Laboratory of Computational Geodynamics, College of Earth and Planetary Sciences, University of Chinese Academy of Sciences, Beijing 100049, China
[5]Key Laboratory of Earth and Planetary Physics, Institute of Geology and Geophysics, Chinese Academy of Sciences, Beijing 100029, China
[6]State Key Laboratory of Lunar and Planetary Sciences, Macau University of Science and Technology, Macau, PR China.
[7]Department of Earth and Planetary Sciences, University of California, Davis, CA 95616, USA
[8]Key Laboratory of Cenozoic Geology and Environment, Institute of Geology and Geophysics, Chinese Academy of Sciences, Beijing 100029, China
[9]Department of Geological Sciences and Geological Engineering, Queen's University, Kingston, K7L 3N6, Canada
[†]These authors contributed equally: Jilian Jiang, Xinyu Zou.

Email: Ross N. Mitchell, ross.mitchell@mail.iggcas.ac.cn; Yigang Zhang, zhangyigang@ucas.ac.cn

This PDF file includes:

- Supporting text
- Figures S1 to S14
- Tables S1 to S4
- SI References

Other supporting materials for this manuscript include the following:

- Datasets 1 to 3

Supporting Information Text

Prudent use of machine learning to recognize S-type zircon

Some problems in recent machine learning work. Recently reported machine learning work trying to recognize S-type zircon (1) seems to be problematic upon closer inspection. After re-implementing the machine learning algorithms used in the previous study, we discovered several issues that could impact the accuracy and reliability of the prediction results for Jack Hills (JH) zircon. Firstly, we noticed that there were linearly related features in the model, which violates the assumption of feature independence. Secondly, there appeared to be a possible incorrect use of the data normalization for the prediction set. Specifically, this involves standardizing the feature values based on the mean and standard deviation of prediction set itself, rather than the training set. This could result in the prediction set being classified into three types, even if it only included one type, leading to erroneous predictions and undermining the accuracy of the results. The correct method is to use the training set statistics for data normalization which ensures that the same scaling factors are applied consistently across both the training and prediction sets and thus ensure accurate and reliable results. Additionally, the model showed signs of overfitting, meaning that it performed better on the training set than on the test set. Overfitting is typically caused by a lack of parameter tuning and can significantly affect the ability of the model to make accurate predictions. Taken together, these problems would cause the prediction results for JH zircon to be completely wrong, and thus they must be addressed before any meaningful conclusions can be drawn from the data. Thus, to ensure accurate and reliable results, in our work particular attention has been given to the precise application of techniques including feature selection (Main Text and *SI Appendix,* Fig. S7), data normalization, and non-overfitting training method (Materials and Methods).

Notably, although our selected 9 elements are independent, Dy and Y still show apparently linearly-correlated relationship, while Lu and Y has modest correlation. To avoid collinearity problems in our experiments, we add an experiment which excludes only Y, turning out F1-score = 0.96 for the test set as well. For this experiment, the fluctuation of S-type proportions for global detrital zircon shows a similar supercontinent-cycle pattern and the same overall trend of the cycles also showing a secular decrease, while the peak of JH S-type zircon becomes slightly lower (Fig. S12).

Our machine learning workflow

Our machine learning workflow went as follows: (i) preprocess original sets to create machine-learning-suitable databases; (ii) train and optimize machine learning models by tuning the hyperparameters of machine learning models to obtain the optimized parameters; (iii) compare the performance of the different machine learning models and select out the best ones that have the highest F1-score; and (iv) predict the types of Jack Hill zircon using the optimized and evaluated machine learning model.

1) Preprocess original datasets to create machine-learning-suitable databases.

Data measurement. Trace element composition of zircon was determined by laser ablation-inductively coupled plasma-mass spectrometry (LA-ICP-MS) employing an Element XR HR-ICP-MS instrument (Thermo Fisher Scientific, Bremen, Germany) coupled to a 193 nm ArF excimer laser system (Geolas HD, Lambda Physik, Göttingen, Germany) at the State Key Laboratory of Lithospheric Evolution (SKLLE), Institute of Geology and Geophysics, Chinese Academy of Sciences (IGGCAS). The approach is similar to that outlined in (2) with isotopes measured using a peak-hopping mode with a laser beam diameter of ~32 μm and 5 Hz repetition rate. The laser energy density is ~4.0 J/cm2. The Element XR is equipped with a “jet-interface”, which is comprised of a jet sample cone, an X-version skimmer cone, and a high-capacity vacuum pump (On Tool Booster 150, Asslar, Germany). This leads to a signal enhancement in laser sampling mode by a factor of 3–5, resulting in an improved detection capability. Helium is employed as the ablation gas to improve the transporting efficiency of ablated aerosols. NIST SRM 612 reference glass is used for calibration material and GJ-1 and 91500 zircon reference materials are analyzed

for data quality control. Silicon (29Si) is used as an internal standard. The resulting data are reduced using the GLITTER program (3). For most trace elements (>0.005 μg/g), the analytical precision (1 RSD) is at ±10%.

Data compilation and filtering. As described details in paragraph 7 of the main text, we compiled zircon data including 297,172 trace element data of 14,535 "clean zircon" from the GEOROC database (https://georoc.eu/georoc/new-start.asp). Database 1 (see corresponding statistical table Table S1 for reference, *n* = 14,535) is designed for calculating the contents and availability of each element in zircon in order to select input features (Fig. 2). Database 2 (see corresponding statistical table Table S3 for reference, *n* = 1,345) is designed for machine learning, and every zircon in it is a typical non-S- or S-type zircon, or an ancient detrital zircon. All the zircon in Database 2 are further classified into the training set, test set, and prediction set.

2) Train and optimize machine learning models by tuning the hyperparameters of machine learning models to obtain the optimized parameters.

Cross-validation. Cross-validation is a method to determine the prediction performance of a machine learning model (4). By using cross-validation, the raw training set is divided into two new parts: new training set and validation set. The new training set is used to train the machine learning models, while the validation set is specifically used to estimate the prediction error of the machine learning model. In this study, we used the method of Leave-One-Out cross-validation (4) to get validation set, that is, only one sample can be selected from training set into the validation set with the remain samples to train the machine learning model in each iteration. For a dataset of n samples, this training iteration is performed with n times. Then, we could figure out the average prediction error of n validation sets, which is the indicator of prediction potential of machine learning models.

Tuning parameters to select ideal parameters for machine learning model. We applied the grid search method (5) to search for the best hyperparameters for machine learning methods, which is to try every combination of parameters for potential training models. The best-performing parameters, which have the best F1-score on the validation set, is determined by applying the method of cross-validation. After selecting the best parameters of machine learning algorithm, we fed all samples of raw training set into the machine learning model to get the final well-trained model. Finally, the well-trained model with definite parameters is able to be applied to predict unknown detrital zircon.

3) Evaluate model by comparing performance of different machine learning models with accuracy and F1-score.

Model Evaluation. Generally, accuracy and F1-score are most common indicators used to evaluate a machine learning model (6). In our experiment, the S-type zircon is the positive samples and non-S-type is the negative samples. First, we denote the number of correct-predicted S-type zircon as *TP*, correct-predicted non-S-type zircon as *FP*, wrong-predicted S-type zircon as *FN*, wrong-predicted non-S-type zircon as *TN*. The accuracy is defined by the ratio of all correct-predicted non-S-type zircon and S-type zircon in the test set, calculated by

$$Accuracy = \frac{TP + TN}{TP + FT + TN + FN} \tag{4}$$

Two indicators *Precision* and *Recall* is designed to observe prediction performance of positive samples. Precision represents the probability of correctly-predicted positive samples among the samples predicted to be positive samples, defined as

$$Precision = \frac{TP}{TP + FP} \tag{5}$$

while recall is the probability of being correctly predicted as a positive sample in the original positive samples, calculated by

$$Recall = \frac{TP}{TP + FN} \tag{6}$$

Higher precision and recall rate show that a machine learning method is better. And F1-score is defined as a balanced score of precision and recall, calculated by the following function

$$F1-score = 2*\frac{Precision*Recall}{Precision+Recall} \quad (7)$$

A good machine learning algorithm usually performs with high precision and high recall, corresponding to high F1-score. Its value is closer to 1 means better performance.

4) Predict the types of Jack Hills zircon using the optimized and evaluated machine learning model. By evaluating and comparing the performance of 11 different machine learning models (details in Materials and Methods) via F1-score and accuracy of low-P S-type zircon we chose the best performed model TSVM.

Caution about the prediction of Hadean zircon based on younger zircon

Lower [Zr] of magma may cause the delayed crystallization of zircon in the Hadean–Archean eons compared to the Phanerozoic (7, 8). This likely lead to the caution about the application of our machine learning method recognizing older S-type zircon based on younger zircon. Low [Zr] in early Earth magmas could indeed reduce overall zircon production, aligning with our observed lower abundances (Fig. 4). Yet, our Jack Hills focus is on S-type proportion, not abundance. That is, when S-type zircon and I-type zircon occur at similar crystallization temperatures (9), lower [Zr] is likely to impose a similar effect on the saturation of zircon in I-type and S-type magmas, and as such, zircon saturation in both zircon types is equally delayed and diminished (7). Therefore, this means that under the same low [Zr] conditions of early Earth, the relative proportion of zircon crystallization in both types of magmas may remain unaffected. But this possible limitation of our machine learning method needs to be addressed by some future studies with the same effects on S-type zircon and other types of zircons.

Also, it is impossible to avoid a possible distinction of higher U content in early magma source between Phanerozoic and Hadean–Archean magma sources. To address this concern, we conducted an additional sensitivity test using the raw Th and U contents to train TSVM model and the results illustrate a very similar trend in the proportion of S-type zircon (Fig. S14). This means that a decay-related historical distinction in Th and U contents would not affect our results.

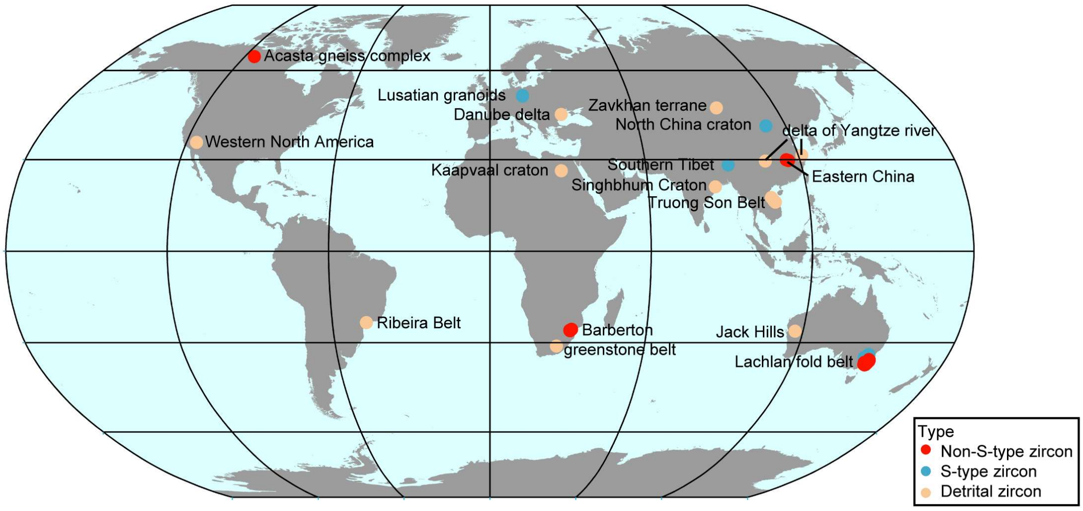

Fig. S1. Simplified geological map of the sample locations of this study and in the literature.

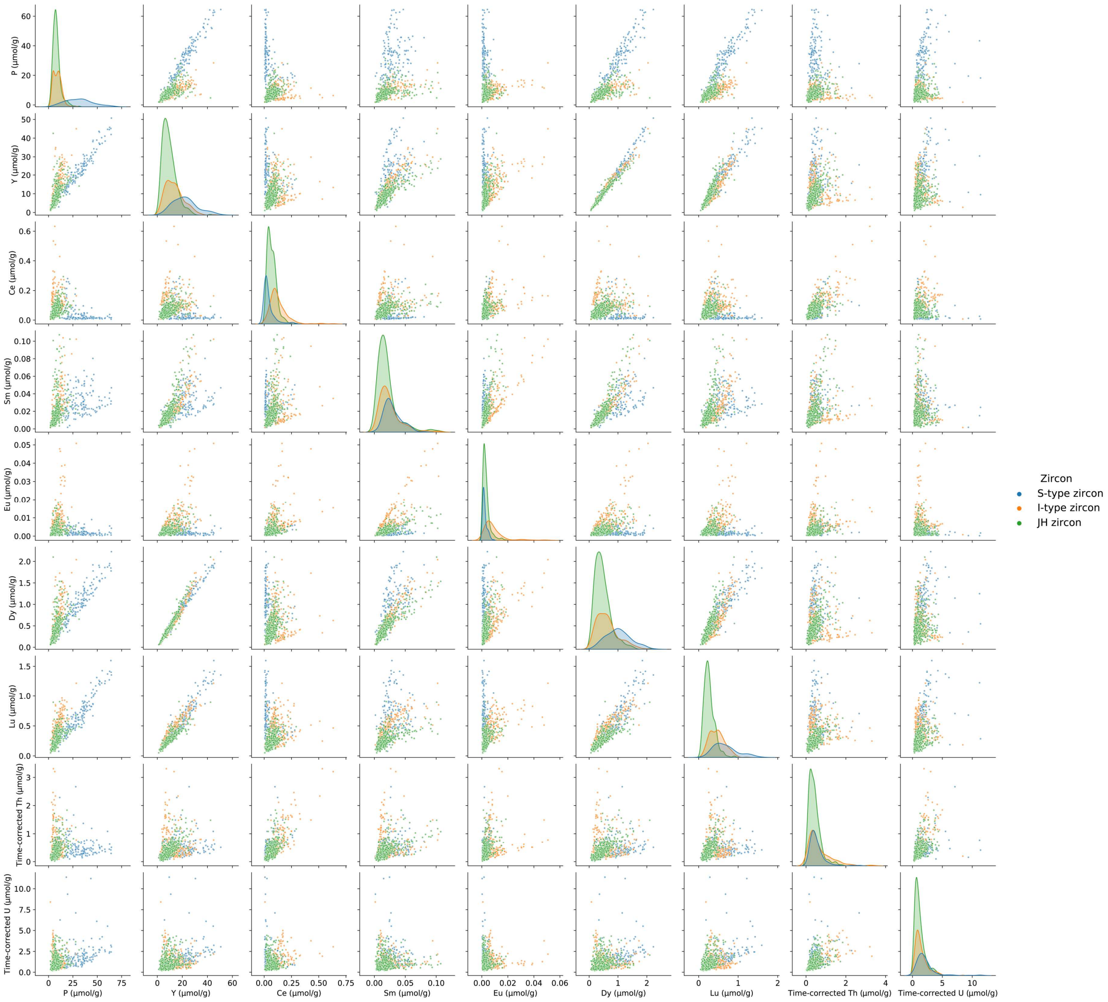

Fig. S2. Distribution and probability density plot by Gaussian Kernel Density Estimation (KDE) of trace element data for Phanerozoic I- and S-type zircon and JH zircon.

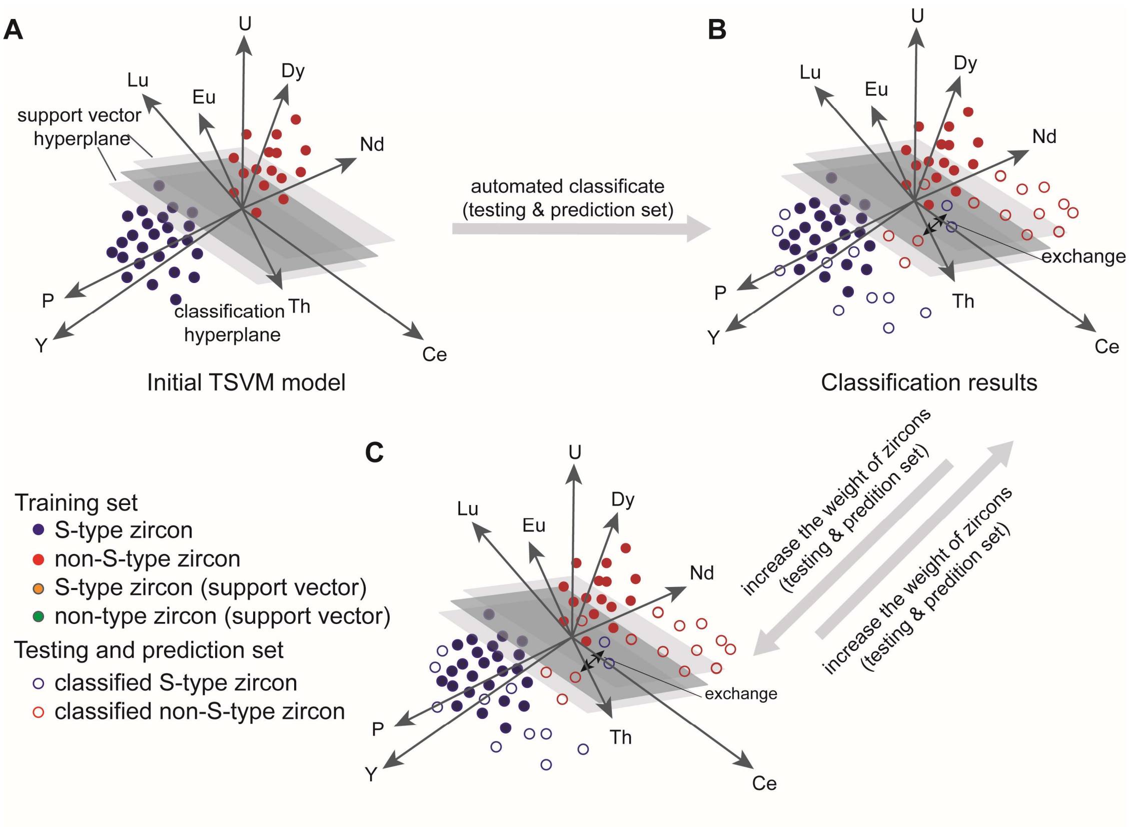

Fig. S3. Schematic diagram showing the principle and workflow of the Transductive Support Vector Machine (TSVM) in high-dimensional space. (A) Initial TSVM model using trace element features in the training set. (B) Exchange zircon between support vectors. (C) Update the TSVM model by using trace element features in the training set, as well as weighted trace element features in the test and prediction sets. Dark grey shows the hyperplane that is used to classify S- and non-S-type zircon, light grey shows the hyperplane defined by support vectors. S-, and non-S-type zircos (training set) are shown as red and blue solid circles, respectively. Classified S- and non-S-type zircon (test and prediction sets) are shown as red and blue empty circles. Support vectors (S- and non-S-type zircon) are shown as green and orange circles. The trace element features used in the TSVM model are shown as arrows (not to scale).

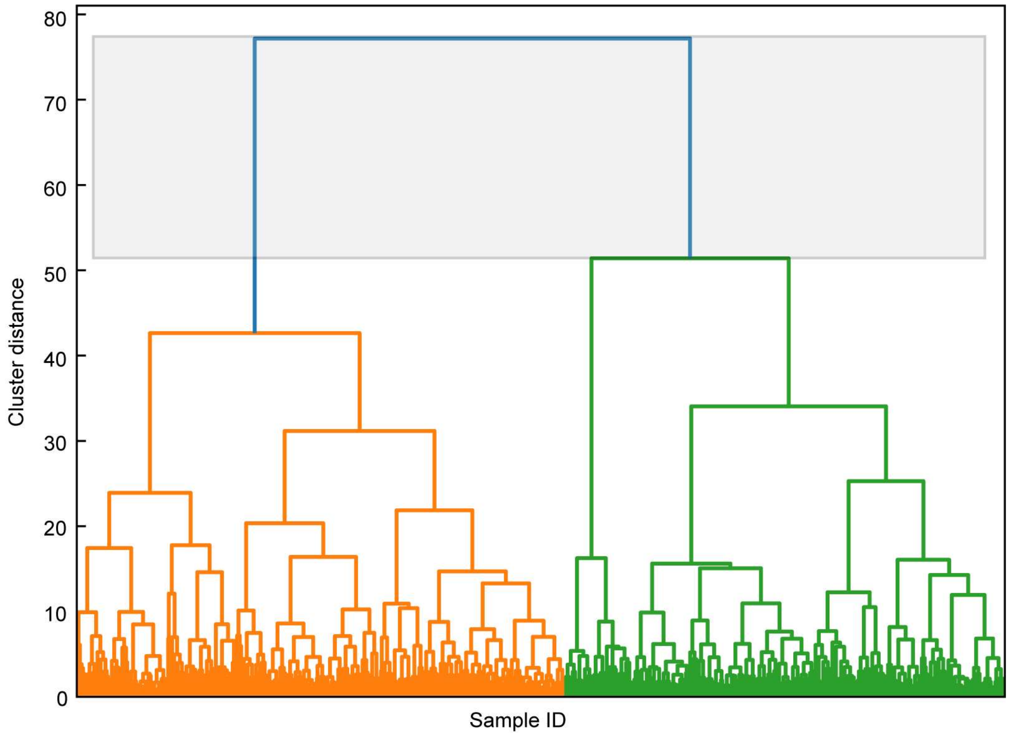

Fig. S4. Cluster tree of all zircon (including 1,345 zircon grains) in the data set. This layout allows for a clear visualization of the hierarchical structure of the data set, with closely related samples or clusters appearing closer together (smaller cluster distance) becoming a new cluster. The *y*-axis cluster distance shows that the distance between different groups or clusters. For instance, if you use a rectangle with cluster distance ranging from 45 to 50 to cut this tree, you will get 3 clusters. The optimal number of clusters will be 2 clusters, which is obtained by the max-height rectangle representing the maximum Euclidean distance between these clusters.

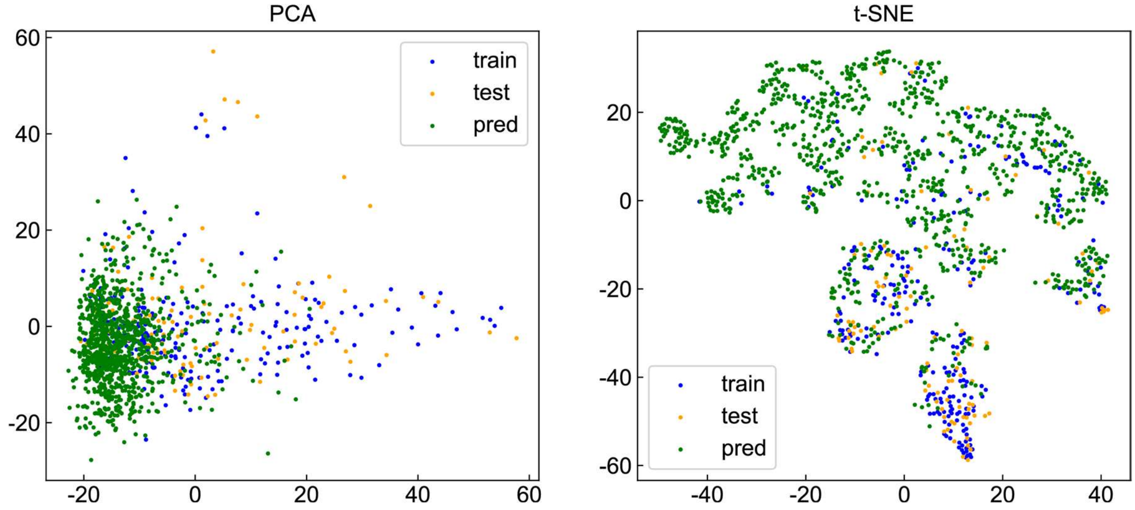

Fig. S5. PCA and t-SNE analysis for the domain of training set, test set, and prediction set. It indicates that the prediction set is located in the domain of training set and test set.

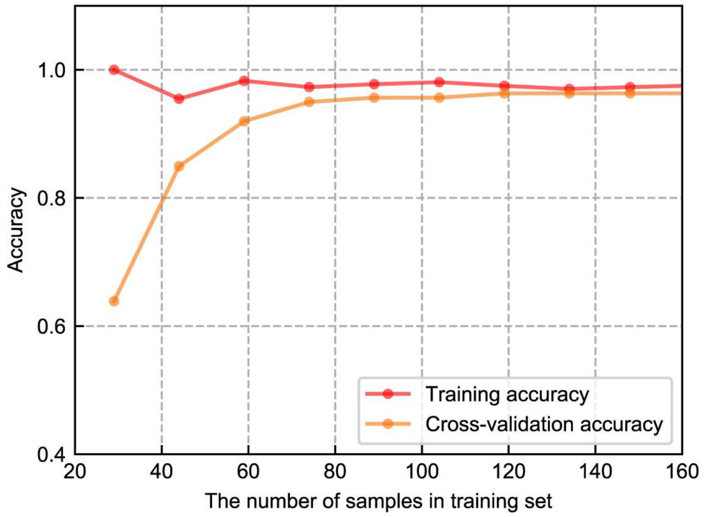

Fig. S6. Learning curve during training of the machine learning algorithm, showing TSVM as an example.

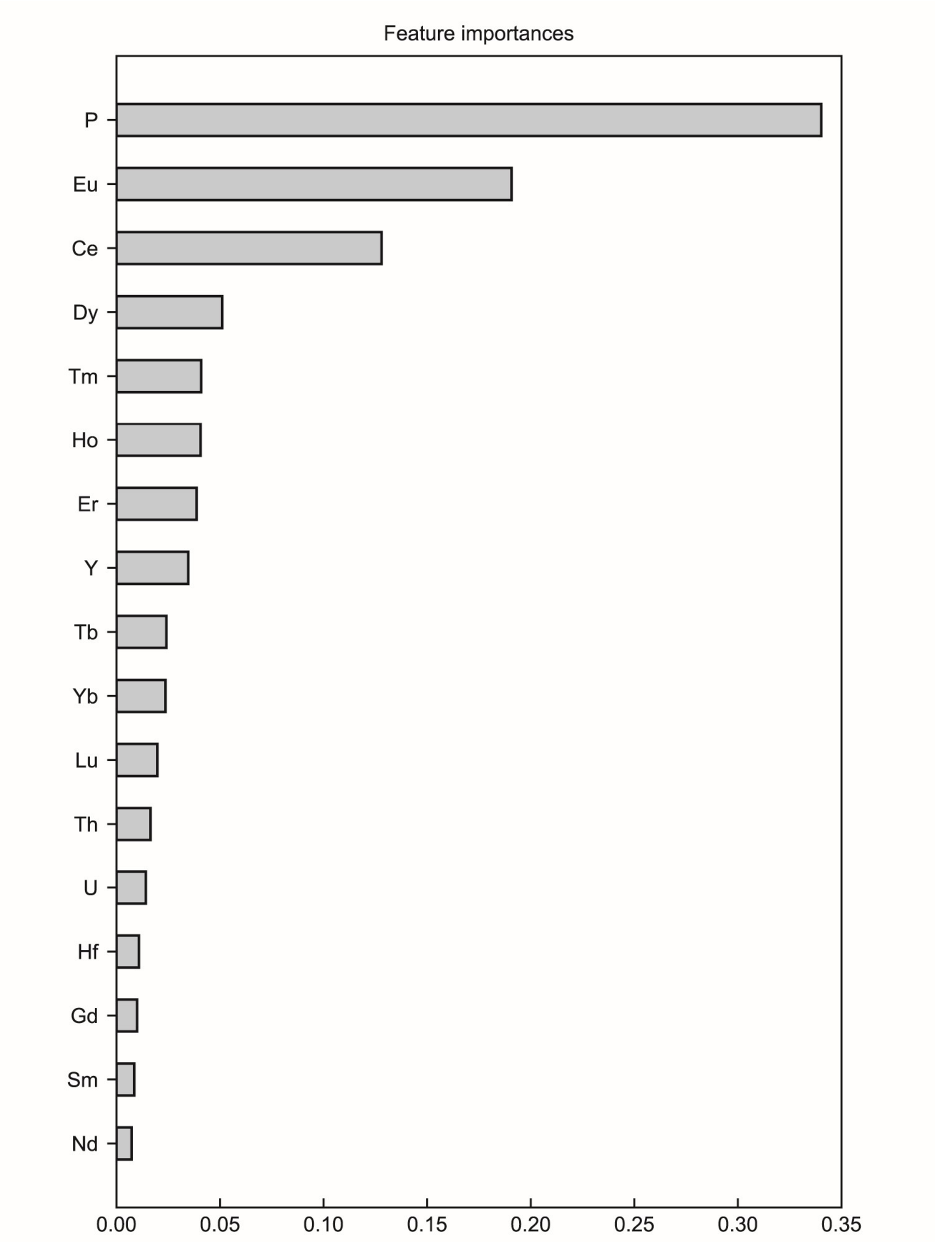

Fig. S7. Importance of 17 elements for classification of non-S-/S-type zircon obtained by applying Random Forest algorithm **(10–12)**.

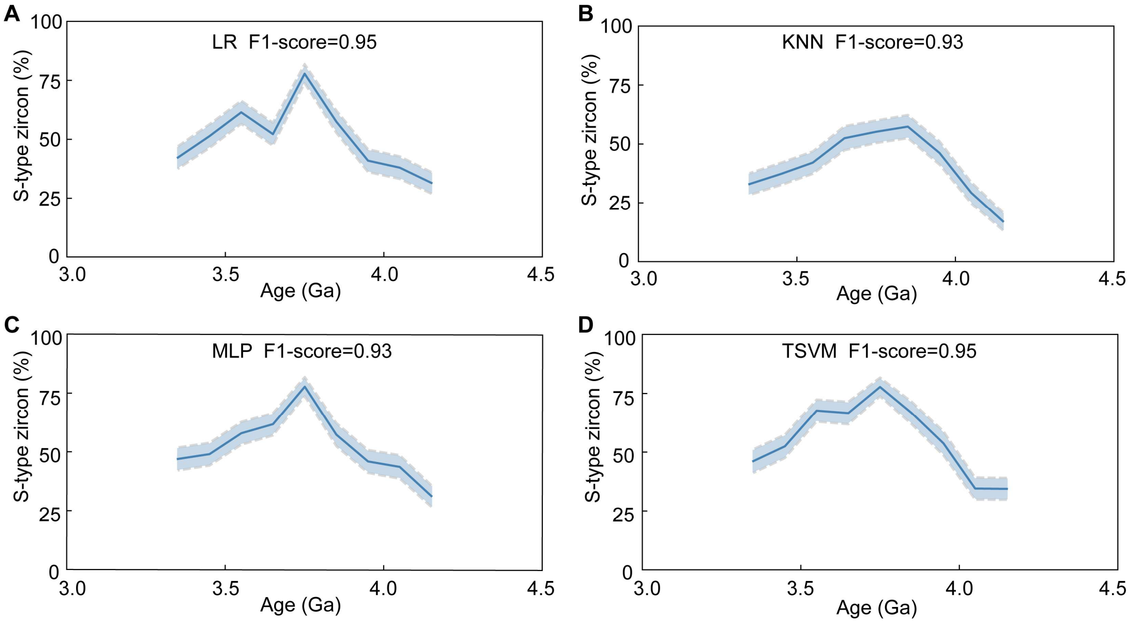

Fig. S8. Sensitivity analysis of the JH zircon proportion regarding different machine learning methods.

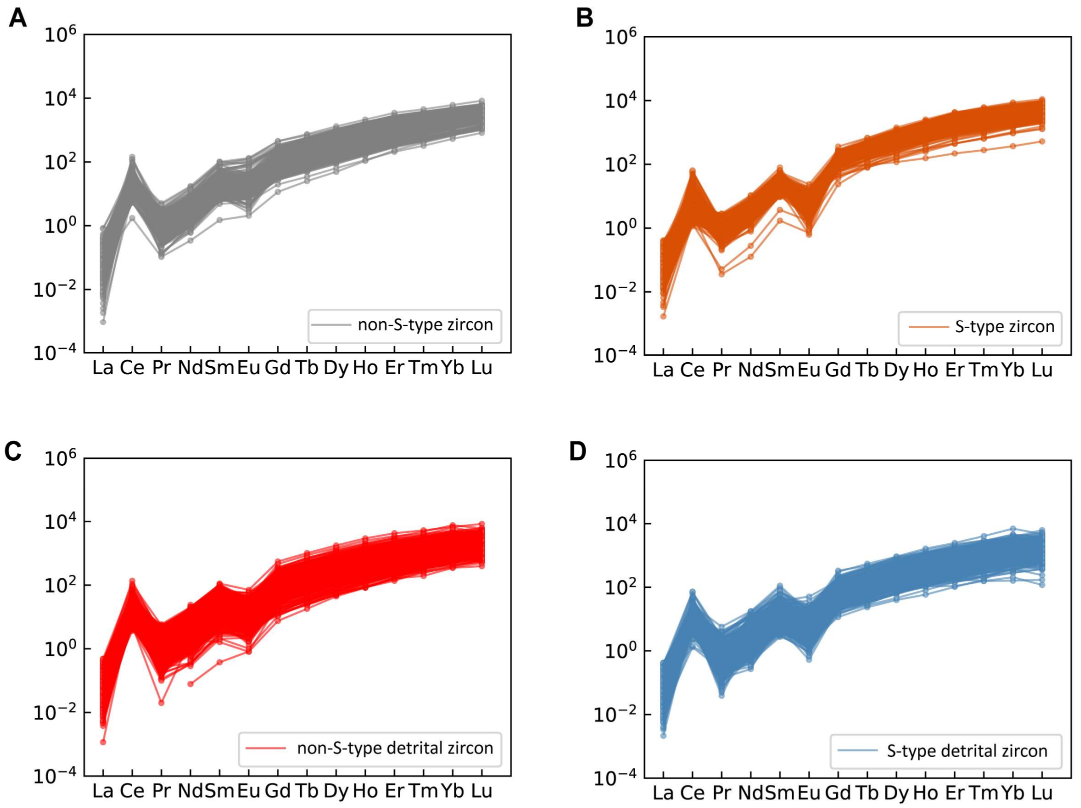

Fig. S9. Chondrite-normalized zircon REE compositions of (A–B) modern non-S- and S-type zircon (C–D) non-S- and S-type detrital zircon predicted by the TSVM algorithm.

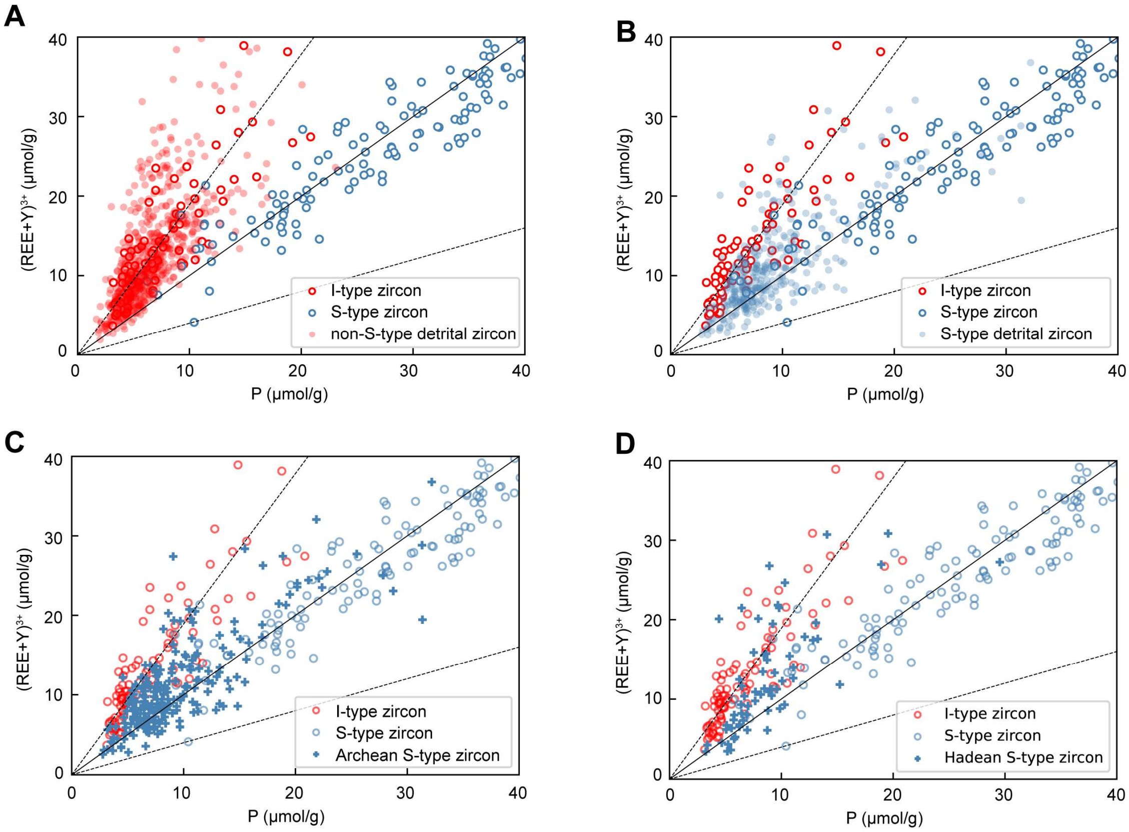

Fig. S10. Trace element distribution of I-, S-type zircon and detrital zircon. More clear distributions from (B) P-REE+Y^{3+} diagram of S-type detrital zircon is shown in (C) P-REE+Y^{3+} diagram of Archean S-type detrital zircon (D) P-REE+Y^{3+} diagram of Hadean S-type detrital zircon. Two dotted lines indicate the "traditional S-type region" constraining all known S-type zircon and the solid line is the xenotime substitution. The solid symbols are detrital zircon, the red symbols are I-type zircon, the blue symbols are S-type zircon.

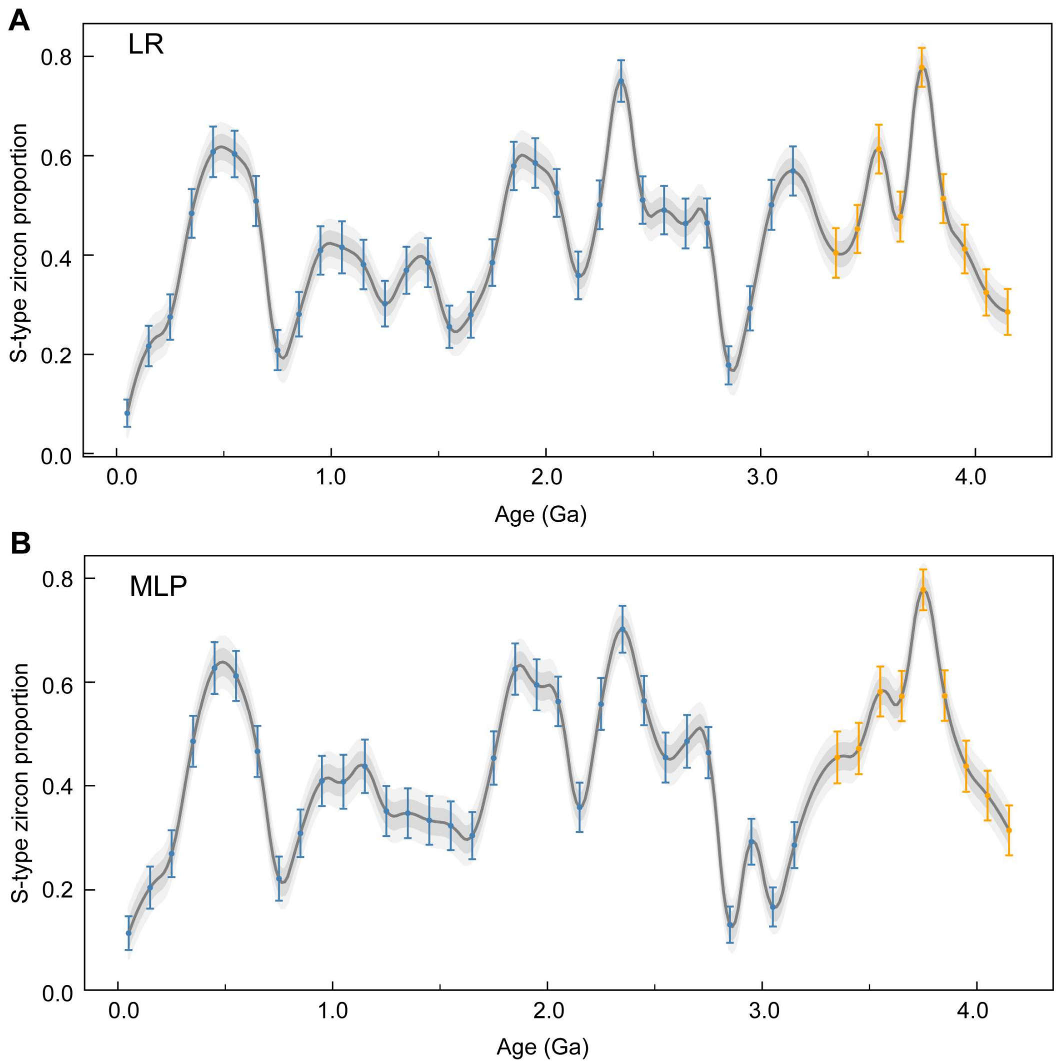

Fig. S11. S-type zircon proportion curves of global detrital zircon based on predicted results from two other machine learning algorithms: (A) Logistic Regression (LR) and (B) Multilayer Perceptron (MLP).

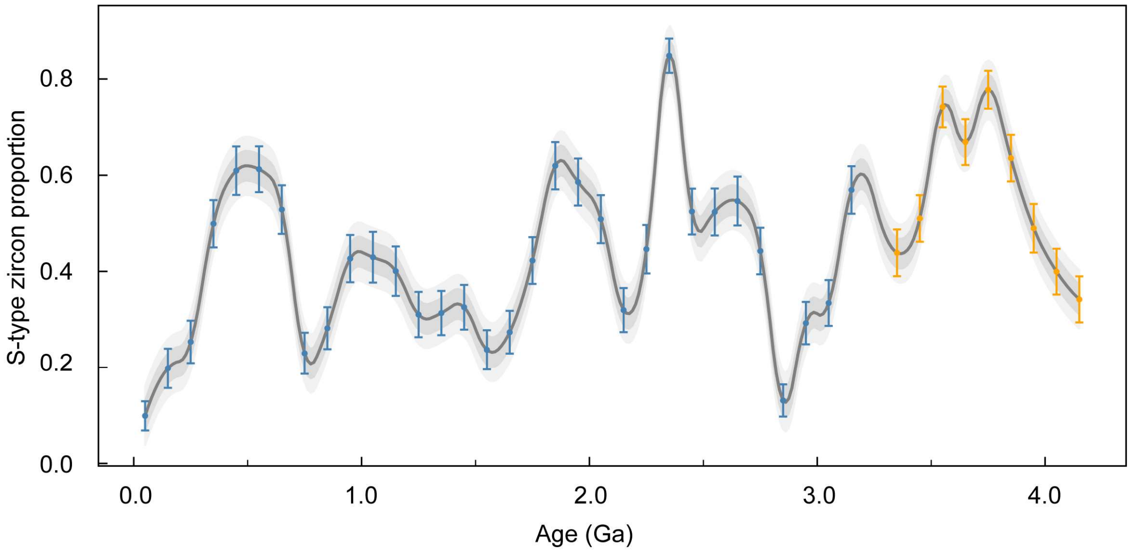

Fig. S12. S-type zircon proportion curves predicted by TSVM using all input features excluding Y.

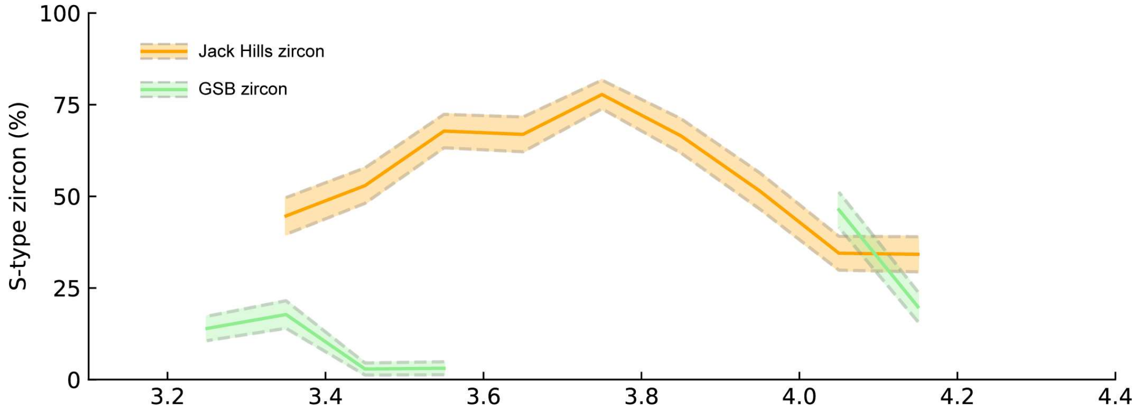

Fig. S13. S-type zircon proportions for the Jack Hills zircon and the Green Sandstone Bed (GSB) zircon (clean zircons filtered by La <1ppm) (8, 13) in the Hadean–early Archean.

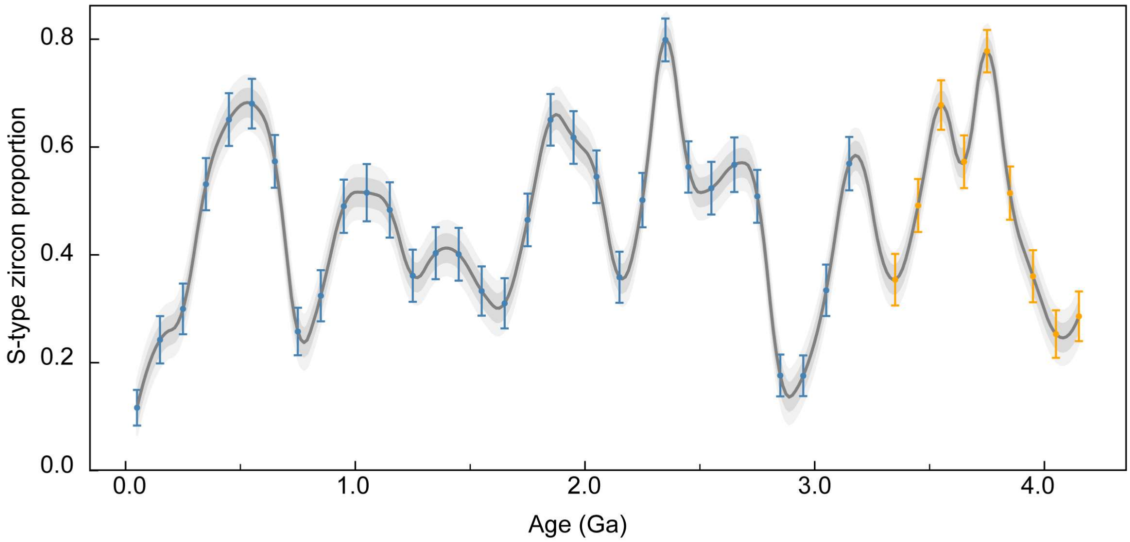

Fig. S14. S-type zircon proportions predicted by TSVM using raw Th and U data (i.e., without time decay-related correction) as input features.

Table S1. Trace element composition and availability of zircon showing statistical analysis data based on Dataset 1. The 297,172 trace element data in Dataset 1 used for statistical analysis are compiled from 14,535 “clean zircon” (13,14) from the GEOROC database. It is designed for calculating the contents and availability of each element in zircon in order to select input features.

Element	Input feature for machine learning	Number of samples	Availability	Median (μmol/g)	Median (mol%)
Hf		11,972	82.37%	56.28	65.56%
Y	√	11,963	82.30%	10.41	12.13%
P	√	3,612	24.85%	8.59	10.01%
U	√	12,479	85.85%	1.00	1.16%
Th	√	12,398	85.30%	0.59	0.68%
Lu		13,419	92.32%	0.35	0.40%
Yb	√	14,418	99.20%	1.80	2.09%
Tm		12,562	86.43%	0.19	0.22%
Er		14,448	99.40%	0.84	0.98%
Ho		13,221	90.96%	0.18	0.21%
Dy	√	14,532	99.98%	0.48	0.56%
Tb		12,909	88.81%	0.04	0.05%
Gd		14,410	99.14%	0.11	0.13%
Eu	√	14,376	98.91%	<0.01	<0.01%
Sm	√	14,462	99.50%	0.02	0.02%
Nd		14,312	98.47%	0.01	0.01%
Pr		11,013	75.77%	<0.01	<0.01%
Ce	√	14,481	99.63%	0.09	0.10%
La		11,078	76.22%	<0.01	<0.01%
Ti		10,302	70.88%	0.17	0.20%
Nb		8,970	61.71%	0.02	0.03%
Ta		7,074	48.67%	0.01	0.01%
Pb		4,434	30.51%	0.10	0.11%
Fe		1,901	13.08%	0.03	0.03%
Ca		1,260	8.67%	0.11	0.13%
Sc		1,934	13.31%	2.61	3.04%

Li		1,403	9.65%	0.12	0.14%
Sr		1,805	12.42%	<0.01	<0.01%
B		894	6.15%	0.01	0.01%
Rb		855	5.88%	<0.01	<0.01%
other 20 elements		2~649	0.01~ 4.46%	<0.01 ~ 0.55 (sum = 1.69)	<0.01% ~ 0.64% (sum = 1.97%)

Table S2. Comparison of the performance of 11 machine learning algorithms on the test set.

Algorithms			Precision	Recall	F1-score	Zircon grains	Total F1-score	F1-score of low-P S-type zircon	Accuracy of low-P S-type zircon
1	Decision Tree	non-S-type zircon	0.91	0.93	0.92	44	0.91	0.88	0.75
		S-type zircon	0.9	0.87	0.89	31			
		Weighted avg	0.91	0.91	0.91	75			
2	Gaussian NB	non-S-type zircon	0.81	1	0.9	44	0.87	0.83	0.375
		S-type zircon	1	0.68	0.81	31			
		Weighted avg	0.89	0.87	0.86	75			
3	Bernoulli	non-S-type zircon	0.83	0.98	0.9	44	0.87	0.83	0.4375
		S-type zircon	0.96	0.71	0.81	31			
		Weighted avg	0.88	0.87	0.86	75			
4	Logistic	non-S-type zircon	0.96	0.98	0.97	44	0.96	0.95	0.875
		S-type zircon	0.97	0.94	0.95	31			
		Weighted avg	0.96	0.96	0.96	75			
5	SVM	non-S-type zircon	0.93	0.95	0.94	44	0.93	0.92	0.8125
		S-type zircon	0.93	0.9	0.92	31			
		Weighted avg	0.93	0.93	0.93	75			
6	KNN	non-S-type zircon	0.92	1	0.96	44	0.95	0.93	0.8125
		S-type zircon	1	0.97	0.93	31			
		Weighted avg	0.95	0.95	0.95	75			
7	MLP	non-S-type zircon	0.93	0.98	0.96	44	0.95	0.93	0.8125
		S-type zircon	0.97	0.9	0.93	31			
		Weighted avg	0.95	0.95	0.95	75			
8	Voting	non-S-type zircon	0.93	0.95	0.94	44	0.93	0.92	0.8125
		S-type zircon	0.93	0.9	0.92	31			
		Weighted avg	0.93	0.93	0.93	75			
9	Bagging	non-S-type zircon	0.93	0.95	0.94	44	0.93	0.92	0.8125
		S-type zircon	0.93	0.9	0.92	31			
		Weighted avg	0.93	0.93	0.93	75			
10	Adaboost	non-S-type zircon	0.9	0.98	0.93	44	0.92	0.90	0.6875
		S-type zircon	0.96	0.84	0.9	31			

		Weighted avg	0.92	0.92	0.92	75			
11	TSVM	non-S-type zircon	0.96	0.98	0.97	44	0.96	0.95	0.875
		S-type zircon	0.97	0.94	0.95	31			
		Weighted avg	0.96	0.96	0.96	75			

Table S3. S-type zircon proportion of 12 locations with detrital zircon. Hadean Jack Hills zircon data (i.e., ages >4 Ga) are noted as bold. The S-type zircon and non-S-type zircon for a data set mean the number of the S-type zircon and non-S-type zircon data. S-type zircon (%) represents the proportion of S-type zircon in a data set.

Location		Split dataset used for machine learning	Zircon	S-type zircon	Non-S-type zircon	S-type zircon (%)
Lachlan Fold Belt, Australia		Training set / Test set	119	79	40	—
South East, China			54	0	54	
Acasta Gneiss Complex, Canada			11	0	11	—
Barberton granite–greenstone terrane, South Africa			115	0	115	
Germany			50	50	0	
Himalaya			16	16	0	
North China Craton			9	9	0	
Total			374	154	220	41.4
Jack Hills, Australia (all)		Prediction set	403	201	202	49.9%
Hadean Jack Hills zircon (4000±80Ma)	(> 4080 Ma)		49	16	33	32.7
	(> 4000 Ma)		**94**	**33**	**61**	**35.1**
	(> 3920 Ma)		122	50	72	50.0
Western Australia			296	72	224	24.3
Zavkhan Terrane, southwestern Mongolia			63	26	37	41.3
Western north America			59	42	17	71.2
Witwatersrand, Kaapvaal craton			34	1	33	2.9
Truong Son Belt, central Vietnam			27	22	5	81.5
Singhbhum Craton, eastern India			21	11	10	52.4
Ribeira Belt, SE, Brazil			15	5	10	33.3

Jiaolai basin, China		15	10	5	66.7
Danube delta, Romania		11	4	7	36.4
Yangtze delta, China		8	2	6	25

Table S4. Bootstrapped averages of S-type Jack Hills zircon proportions through time.

Age (Ma)	S-type zircon proportion	1 sigma error	Number of zircon	Figure 4B
4350	0		2	
4250	0.50		2	
4150	0.34	0.05	35	√
4050	0.35	0.05	55	√
3950	0.54	0.05	39	√
3850	0.67	0.04	33	√
3750	0.78	0.05	9	√
3650	0.67	0.05	21	√
3550	0.71	0.05	31	√
3450	0.52	0.05	51	√
3350	0.44	0.05	121	√
3250	0.33	0.05	3	
3150			0	
3050	0		1	

SI References

1. S. Zhong, S. Li, Y. Liu, P. A. Cawood, R. Seltmann, I-type and S-type granites in the Earth's earliest continental crust. *Commun Earth Environ* **4**, 61 (2023).
2. P. Holden, *et al.*, Mass-spectrometric mining of Hadean zircons by automated SHRIMP multi-collector and single-collector U/Pb zircon age dating: The first 100,000 grains. *International Journal of Mass Spectrometry* **286**, 53–63 (2009).
3. G. W. L, GLITTER : Data reduction software for laser ablation ICP-MS. *Laser Ablation ICP-MS in the Earth Sciences : Current practices and outstanding issues* 308–311 (2008).
4. M. W. Browne, Cross-Validation Methods. *Journal of Mathematical Psychology* **44**, 108–132 (2000).
5. H. A. Fayed, A. F. Atiya, Speed up grid-search for parameter selection of support vector machines. *Applied Soft Computing* **80**, 202–210 (2019).
6. R. Yacouby, D. Axman, Probabilistic Extension of Precision, Recall, and F1 Score for More Thorough Evaluation of Classification Models in *Proceedings of the First Workshop on Evaluation and Comparison of NLP Systems*, (Association for Computational Linguistics, 2020), pp. 79–91.
7. C. B. Keller, P. Boehnke, B. Schoene, Temporal variation in relative zircon abundance throughout Earth history. *Geochem. Persp. Let.* 179–189 (2017). https://doi.org/10.7185/geochemlet.1721.
8. N. Drabon, *et al.*, Heterogeneous Hadean crust with ambient mantle affinity recorded in detrital zircons of the Green Sandstone Bed, South Africa. *Proceedings of the National Academy of Sciences* **118**, e2004370118 (2021).
9. M. R. Ackerson, D. Trail, J. Buettner, Emergence of peraluminous crustal magmas and implications for the early Earth. *Geochem. Persp. Let.* **17**, 50–54 (2021).
10. Y. Zhao, Y. Zhang, M. Geng, J. Jiang, X. Zou, Involvement of Slab-Derived Fluid in the Generation of Cenozoic Basalts in Northeast China Inferred From Machine Learning. *Geophys. Res. Lett.* **46**, 5234–5242 (2019).
11. K. Itano, H. Sawada, Revisiting the Geochemical Classification of Zircon Source Rocks Using a Machine Learning Approach. *Math Geosci* 1–22 (2024). https://doi.org/10.1007/s11004-023-10128-z.
12. L. Breiman, Random Forests. *Machine Learning* **45**, 5–32 (2001).
13. N. Drabon, H. M. Kirkpatrick, G. R. Byerly, J. L. Wooden, Trace elements in zircon record changing magmatic processes and the multi-stage build-up of Archean proto-continental crust. *Geochimica et Cosmochimica Acta* **373**, 136–150 (2024).
